# Robustness analysis on interspecies interaction network for iron and glucose competition between *Candida albicans *and zebrafish during infection

**DOI:** 10.1186/1752-0509-8-S5-S6

**Published:** 2014-12-12

**Authors:** Che Lin, Chin-Nan Lin, Yu-Chao Wang, Fang-Yu Liu, Yu-Wen Chien, Yung-Jen Chuang, Chung-Yu Lan, Wen-Ping Hsieh, Bor-Sen Chen

**Affiliations:** 1 Institute of Communication Engineering, National TsingHua University, Hsinchu 30013, Taiwan; 2 Department of Electrical Engineering, National TsingHua University, Hsinchu 30013, Taiwan; 3 Institute of Biomedical Informatics, National Yang-Ming University, Taipei, Taiwan; 4 Department of Medical Science and Institute of Bioinformatics and Structural Biology, National TsingHua University, Hsinchu 30013, Taiwan; 5 Department of Life Science and Institute of Molecular and Cellular Biology, National TsingHua University, Hsinchu 30013, Taiwan; 6 Institute of Statistics, National TsingHua University, Hsinchu 30013, Taiwan

## Abstract

*Candida albicans *has emerged as an important model organism for the study of infectious disease. Using high-throughput simultaneously quantified time-course transcriptomics, this study constructed host-pathogen interspecies interaction networks between *C. albicans *and zebrafish during the adhesion, invasion, and damage stages. Given that iron and glucose have been identified as crucial resources required during the infection process between *C. albicans *and zebrafish, we focused on the construction of the interspecies networks associated with them. Furthermore, a randomization technique was proposed to identify differentially regulated proteins that are statistically eminent for the three infection stages. The behaviors of the highly connected or differentially regulated proteins identified from the resulting networks were further investigated.

"Robustness" is an important system property that measures the ability of the system tolerating the intrinsic perturbations in a dynamic network. This characteristic provides a systematic and quantitative view to elucidate the dynamics of iron and glucose competition in terms of the interspecies interaction networks. Here, we further estimated the robustness of our constructed interspecies interaction networks for the three infection stages.

The constructed networks and robustness analysis provided significant insight into dynamic interactions related to iron and glucose competition during infection and enabled us to quantify the system's intrinsic perturbation tolerance ability during iron and glucose competition throughout the three infection stages. Moreover, the networks also assist in elucidating the offensive and defensive mechanisms of C. albicans and zebrafish during their competition for iron and glucose. Our proposed method can be easily extended to identify other such networks involved in the competition for essential resources during infection.

## Introduction

According to the World Health Organization, infectious disease remains the leading cause of death for the youth worldwide and people living in low-income countries [1]. As one of the most commonly found commensal and opportunistic fungal pathogens in humans, *Candida albicans *has become an important model organism for the study of infectious disease. Many factors such as adherence, morphological changes, and secreting hydrolytic enzymes have been shown to be important virulence factors and are linked to the pathogenesis of *C. albicans *[2,3]. As a result, understanding the complicated and intertwined mechanisms of *C. albicans *pathogenesis and its interaction with the host has emerged as an important area of study.

The use of zebrafish as model host in studies of infectious diseases caused by bacteria or viruses is increasing. The genetics, anatomical structure, and physiology of zebrafish are similar to those of mammals, yet their reproductive rate is much higher and the species requires relatively low maintenance costs [4]. In addition, zebrafish has both innate and adaptive immune functions, making them particularly suitable as a model organism for investigating infection [4-6].

"Robustness" is an important system property that measures the ability of the system tolerating the intrinsic perturbations in a dynamic network [7]. In any system, perturbation and uncertainty are inevitable and may render the system unstable. The robustness of a system has become an important topic of interest in industrial design since 1970s [8]. In a biological system, such uncertainty may arise from the intrinsic perturbation of living organisms often caused by mutation, thermal fluctuation and other process noise [9]. These intrinsic perturbations might render the living organisms physiology mechanisms uncoordinated such as metabolic imbalance, hypothermia or cytopathogenesis and death eventually. Therefore, robustness, the ability for living organisms to maintain against intrinsic perturbations, is viewed as a key property for a biological system [10].

This study aims to understand the complicated offensive and defensive mechanisms of *C. albicans *and zebrafish during the infection process by constructing interspecies interaction networks between the pathogen and the host, as well as the estimation of the robustness of the networks through the quantified time-course transcriptomics. In our previous report [11], we constructed the entire interspecies protein interaction network between *C. albicans *and zebrafish. Here, instead, we focused on the construction of interspecies interaction networks that are associated with iron and glucose, two of the most crucial resources that are competed for during the infection of zebrafish by *C. albicans*. We further constructed the iron- and glucose competition interspecies networks for three infection stages, i.e., the adhesion, invasion, and damage stages. Proteins that were highly connected or differentially regulated among different infection stages for both the iron and glucose interaction networks were also identified. Finally, we estimated the robustness of each interspecies network that helps us elucidate the dynamics of iron and glucose competition during the infection process. These analyses provided insight into the dynamic behaviors of resource competition between *C. albicans *and zebrafish and highlighted significant proteins that may play important roles during infection. Based on the developed networks, we were able to quantify the progression of iron and glucose competition throughout the three infection stages. Furthermore, the constructed interspecies networks with robustness estimation help elucidate the offensive and defensive mechanisms of *C. albicans *and zebrafish during their competition for iron and glucose.

In-depth understanding of the dynamic host-pathogen interspecies interaction networks of the pathogen during infection is essential in the development of new anti-fungal drugs and the improvement of corresponding medical therapy. Our proposed method and *in silico *experiments could lay the foundation for the development of new therapeutic strategies against infectious diseases.

## Materials and methods

### Overview of construction of host-pathogen interspecies interaction networks

**Figure 1:**
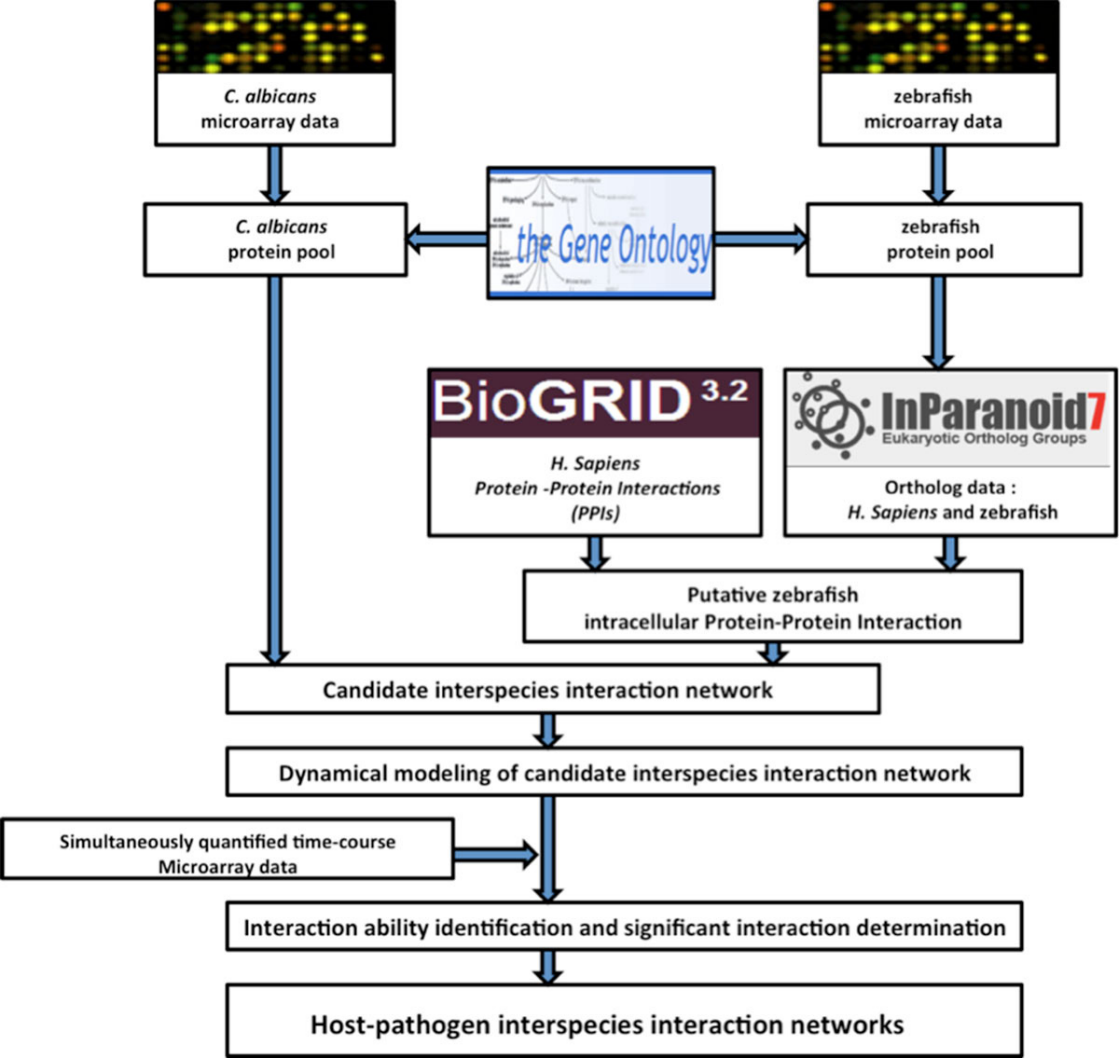
**Flowchart of host-pathogen interspecies interaction network construction**. The Gene Ontology database was used to select *C. albicans *and zebrafish protein pools. Protein-protein interaction (PPI) data from the BioGRID database, ortholog information from InParanoid, and simultaneously quantified time-course microarray data for both *C. albicans *and zebrafish during *C. albicans*-zebrafish interactions were used for interspecies protein interaction network construction. Note that to construct our interspecies networks, we substituted all gene expression profiles for protein activity levels since large-scale proteomic data is still not widely available.

Our method for the construction of host-pathogen interspecies interaction networks included three major steps: (i) data selection, (ii) protein pools selection, and (iii) construction of networks. The proposed interspecies interaction network construction is shown in Figure 1. To study the competition for iron and glucose between *C. albicans *and zebrafish during infection, we first selected suitable protein pools for *C. albicans *and zebrafish that were associated with these important resources. Putative interspecies interaction networks were then identified based on known protein-protein interactions (PPIs) from existing databases, our proposed dynamic model for indirect interspecies interactions between the two organisms, and simultaneously quantified microarray data for *C. albicans *and zebrafish during infection. Significant interspecies protein interactions between *C. albicans *and zebrafish among these candidates were identified by removing false-positive interactions using model order selection techniques. Statistically significant proteins were furthermore obtained by comparison of the developed network with networks constructed using microarray data of zebrafish target proteins randomized across time-points.

### Dataset mining and integration

Four types of data were used in our method: (i) gene ontology annotation information, (ii) simultaneously quantified time-course microarray gene expression data, (iii) protein-protein interaction data from zebrafish, and (iv) ortholog data between zebrafish and *H. sapiens*. Annotations for *C. albicans *and zebrafish proteins were obtained from the Gene Ontology (GO) database (http://www.geneontology.org/) [12]. This database was used to select the *C. albicans *and zebrafish protein pools as described in the following section (protein pools selection). Genome-wide simultaneously quantified microarray data were obtained from the GEO database (GSE32119). Experiments were performed to obtain *in vivo *genome-wide simultaneously quantified time-course gene expression profiles for both *C. albicans *and zebrafish during *C. albicans *infection of zebrafish. Wild-type zebrafish were injected with *C. albicans *cells and gene expressions for both *C. albicans *and zebrafish were monitored at nine time-points (0.5, 1, 2, 4, 6, 8, 12, 16, and 18 hours post-injection [hpi]) using three replicates. Based on histological analysis by Chen *et al*. [13], we also divided the microarray data from nine time-points into three overlapping stages (adhesion stage: 0.5, 1, 2, 4 hpi; invasion stage: 2, 4, 6, 8, 12 hpi; damage stage: 6, 8, 12, 16, 18 hpi) for both *C. albicans *and zebrafish. Ortholog data between zebrafish and *H. sapiens *were obtained from the InParanoid database (http://inparanoid.sbc.su.se/) [14]. These data were also essential for selecting the zebrafish protein pools. Protein-protein interaction data of *H. sapiens *were extracted from the Biological General Repository for Interaction Datasets (BioGRID; http://thebiogrid.org/) [15].

### Protein pools selection

In this study, we focused on the construction of interspecies protein interaction networks that were associated with iron and glucose to investigate the competition between *C. albicans *and zebrafish for these important resources during infection. As a result, our target protein pools were selected via a function-based selection method. For *C. albicans*, the proteins that were associated with both GO terms of virulence and iron were selected and defined as the iron-related virulence protein pool, while those associated with GO terms of virulence and glucose were defined as the glucose-related virulence protein pool. The corresponding target protein pools for zebrafish were similarly defined as the iron-related and glucose-related immune protein pools. Since few annotations were available for zebrafish, functional annotations for *H. sapiens *proteins were used to infer possible functional annotations of zebrafish with the help of ortholog data; i.e., if a *H. sapiens *protein was associated with immunity and had an orthologous protein in zebrafish, we inferred that the zebrafish protein was also immune-related.

For those *C. albicans *and zebrafish proteins that were selected based on functional annotations, one-way analysis of variance (ANOVA) was applied on the gene expression profiles in our time-course microarray data that correspond to these proteins to further select differentially expressed proteins. The null hypothesis of ANOVA assumed that the average expression level of a gene would be the same at every time point. Proteins from these pools were excluded if the corresponding *p*-values were < 0.01. The selected protein pools are shown in Table 1 and were used to construct the iron- and glucose competition interspecies networks.

**Table 1:** Functional protein pool list.

Protein pool	Proteins symbol
*C. albicans* iron-related virulence proteins	*asc1, bgl2, cat1, hem3, rbt4, rfg1, ppt1, ftr2, nag3, phr2*,
	*yhb1, bud2, erg3, gcs1, int1, ddr48, fre10, hap43, hmx1, sap10*,
	*sit1, ftr1, ccc2, cph1, cyr1, dfg16, efg1, hog1, mnn2, rim101*,
	*snf7, sod1, tup1, vps28, gcn4, sod2, als3, ssn6, tpk2, tpk1*,
	*mig1*

Zebrafish iron-related immune proteins	*alas2, atp6v1h, atp7a, ba2, bcl2, calrl2, cyba*,
	*cybb, ercc2, fech, flvcr1, glrx5, hamp1, hif1ab*,
	*hmox1, hpx, jak2a, jak2b, jmjd6, mb, ncf1*,
	*ndfip1, nos2a, nos2b, rsad2, slc11a2, slc25a37, slc40a1*,
	*sod1, sod2, src, tfr1a*

*C. albicans* glucose-related virulence proteins	*cdc19, cdc24, cek1, tps1, gpr1, tps2, spt3, snf1, gpa2, rim8*,
	*cst20, ino1, fba1, hsl1, cnh1, cdc42, gsc1, fbp1, asc1, itr1*,
	*rim20, tpk2, hgt4, hxk1, cdc14, orf19.6739, orf19.7670*

Zebrafish glucose-related immune proteins	*angpt2, arg2, bad, bpgm, cyba, dbh, edn1*,
	*egr1, fgf21, foxo3a, gpia, gpib, hdac4, hif1ab*,
	*hprt1, ins, lrp5, onecut1, onecutl, pgm3, pla2g1b*,
	*pparg, prkcq, ptenb, rpl11, slc11a2, slc16a3, slc3a2*,
	*slc7a6, src, thbs1, tnfa, tnfb, yes1*

Proteins used to construct iron- and glucose competition interspecies networks are selected from C. albicans and zebrafish based on function annotation with ANOVA-selection. They can be classified into four groups: 1. C. albicans iron-related 2. zebrafish iron-related 3. C. albicans glucose-related and 4. zebrafish glucose-related.

### Construction of the host-pathogen interspecies interaction networks

Interactions between host and pathogen are generally complicated and not easily delineated, making it difficult to identify appropriate stochastic dynamic models to describe them. To determine zebrafish target proteins in this study, we examined potential interactions of *C. albicans *proteins with zebrafish proteins in the selected protein pools using available PPI information.

Due to extremely low coverage of the zebrafish interactomes, ortholog-based PPI prediction was used to infer the potential PPIs within zebrafish. The PPI data of H. sapiens from BioGRID and the ortholog information among these species from the InParanoid database were used to infer the potential intracellular PPIs as proposed in our previous work [16]. We believe that although such cross-species information can affect our candidate networks, the final constructed networks should be more robust against such inaccuracy since actual time-course transcriptome and dynamic modeling were used to prune this candidate model. Once more PPI information become available for zebrafish, the accuracy of our constructed networks can be further improved.

Note that the networks constructed in this manner are biased since known PPI information is used but we believe that such bias is in fact beneficial for the accuracy of the constructed networks. If no such prior information is used, we have to assume that all nodes (proteins) are potentially interactive with all other nodes (proteins). In this case, we believe that there will be more false-positive interactions than the current methodology.

We also assumed that each target protein in the zebrafish iron-related immune protein pool was potentially affected by all proteins in the *C. albicans *iron-related virulence protein pool (analogously for glucose-related proteins) since there is no existing prior information available for interspecies interactions. The host-pathogen iron interspecies interaction network and the host-pathogen glucose interspecies interaction network were constructed based on these assumptions.

The dynamic model for the  k-th zebrafish target protein in the host-pathogen interaction network can be described as:

(1) $$p_{k}\left[t+1\right]=p_{k}\left[t\right]+\sum_{j=1}^{J_{k}}c_{kj}g_{j}\left[t\right]-\kappa_{k}p_{k}\left[t\right]+\sum_{m=1}^{M_{k}}b_{km}p_{k}\left[t\right]p_{m}\left[t\right]+q_{k}+n_{k}\left[t\right]$$

where$p_{k}\left[t\right]$ represents the protein activity level for the  k-th zebrafish target protein at time  t, $g_{j}\left[t\right]$ represents the protein activity level of the  j*-*th *C. albicans *protein that might potentially affect the  k-th zebrafish target protein, and $c_{kj}$ denotes the corresponding interspecies interaction ability between them. We denote $J_{k}$ as the number of potentially regulatory proteins from *C. albicans*, $M_{k}$ as the number of protein-protein interactions in zebrafish for the  k-th target protein, $\kappa_{k}$ as the degradation effect for the  k-th zebrafish target protein, $p_{m}\left[t\right]$ as the protein activity level for the  m-th zebrafish protein that can potentially interact with the  k-th target protein, and $b_{km}$ as the corresponding interaction ability between them. The basal level is denoted by $q_{k}$, and the stochastic noise due to model uncertainty and fluctuation of the microarray data is represented by $n_{k}\left[t\right].$

Compared with our previously developed dynamic network model for single-species network construction [16], it should be noted that in addition to the single-species protein-protein interaction captured by the term associated with the interaction ability $b_{km}$, interspecies interactions are captured by the term associated with the interaction ability $c_{kj}$. Indirect interspecies interactions are complicated in nature. To create a tractable model for further analysis, we chose a linear approximation to capture the nonlinear effect in $c_{kj}$. Since $c_{kj}$ is only used to determine whether the indirect interaction exists and its exact value is never used, such an approximation should provide an initial understanding of an interspecies interaction network. In the Results section, we provide evidence from the literature for the identified proteins obtained from our networks to validate these assumptions and approximations.

Since large-scale proteomic data is still not widely available, we substituted all gene expression profiles for protein activity levels. It was reported that gene expression correlates well with distinct proteins for zebrafish, with several categories of genes as exceptions [17]. The interactive parameters for the proposed dynamic model described above can thus be identified using simultaneously quantified time-course microarray data. For this purpose, equation (1) can be rewritten in regression form as follows:

(2) $$p_{k}\left[t+1\right]=\left[g_{1}\left[t\right]\ldots g_{J_{k}}\left[t\right]p_{k}\left[t\right]p_{k}\left[t\right]p_{1}\left[t\right]\ldots p_{k}\left[t\right]p_{M_{k}}\left[t\right]1\right]\left[\begin{array}{c} C_{k1}\\ \vdots \\ C_{k}J_{k}\\ 1-\kappa_{k}\\ \begin{array}{c} \begin{array}{c} b_{k1}\\ \vdots \\ b_{k_{Mk}} \end{array}\\ q_{k} \end{array} \end{array}\right]+n_{k}\left[t\right]=\psi_{k}\left[t\right]\eta_{k}+n_{k}\left[t\right]$$

where $\psi_{k}\left[t\right]$ denotes the regression vector and $\eta_{k}$ denotes the parameter vector for the  k-th zebrafish target protein, which is to be estimated. We used the cubic spline method to interpolate extra time-points within the simultaneously quantified time-course microarray data to avoid over-fitting in the parameter estimation process. Note that equation (2) for different time-points can further be rearranged as:

(3) $$\left[\begin{array}{c} \begin{array}{c} p_{k}\left[t_{2}\right]\\ p_{k}\left[t_{3}\right] \end{array}\\ \vdots \\ p_{k}\left[t_{L}\right] \end{array}\right]=\left[\begin{array}{c} \psi_{k}\left[t_{1}\right]\\ \psi_{k}\left[t_{2}\right]\\ \vdots \\ \psi_{k}\left[t_{L-1}\right] \end{array}\right]\eta_{k}+\left[\begin{array}{c} n_{k}\left[t_{1}\right]\\ n_{k}\left[t_{2}\right]\\ \vdots \\ n_{k}\left[t_{L-1}\right] \end{array}\right]$$

Defining the notations $P_{k}={\left[p_{k}\left[t_{2}\right]\cdots p_{k}\left[t_{L}\right]\right]}^{T},\psi_{k}={\left[\psi_{k}\left[t_{1}\right]\cdots \psi_{k}\left[t_{L-1}\right]\right]}^{T}$ and $\Omega_{k}={\left[n_{k}\left[t_{1}\right]\cdots n_{k}\left[t_{L-1}\right]\right]}^{T}$, equation (3) can then be rewritten as:

(4) $$P_{k}=\psi_{k}\eta_{k}+{\text{Ω}}_{k}$$

In the dynamic model of host-pathogen interspecies interaction networks, the basal level $q_{k}$ should always be greater than or equal to zero since the protein activity level in the simultaneously quantified time-course microarray data is always non-negative. Therefore, the parameter estimation problem becomes a constrained least-square problem:

(5) $$min||\frac{1}{2}\psi_{k}\eta_{k}-P_{k}{||}_{2}^{2}s.t.A\eta_{k}\leq 0$$

where $A=\left[00\cdots -1\right].$ We focused only on parameters that represent host-pathogen interspecies interactions, i.e., $c_{kj}$. After the parameters were identified, Akaike's Information Criterion (*AIC*) was used to select significant *C. albicans-*zebrafish interactions [18]. *AIC *can be expressed mathematically as:

(6) $$AIC\left(M_{k}\right)=2log\in_{k}+\frac{2M_{k}}{L}$$

where $\in_{k}=\psi_{k}\eta_{k}-P_{k}$, $M_{k}$ denotes the number of estimated parameters and  L denotes the number of the samples used to estimate the parameters. The *AIC *includes both the estimated residual error and model complexity in one statistic. The *AIC *value increases as the number of parameters increases and decreases as the variance of the residual error decreases. Since the variance of the residual error may decrease with increasing number of parameters, minimizing the *AIC *value captures a tradeoff between estimation accuracy and model complexity. Appropriate model order and significant regulations can be determined by ranking models by increasing *AIC*. Host-pathogen interspecies interaction networks between *C. albicans *and zebrafish were constructed based on these criteria. We believe that this is a crucial step that improves the accuracy of our constructed networks significantly since PPI information from BioGRID includes all possible interactions in *all conditions*. This creates many false-positive interactions that need to be and can be excluded based on the actual time-course transcriptome and AIC model order selection in the *current condition*.

### Identification of differentially regulated proteins via randomization techniques

**Figure 2:**
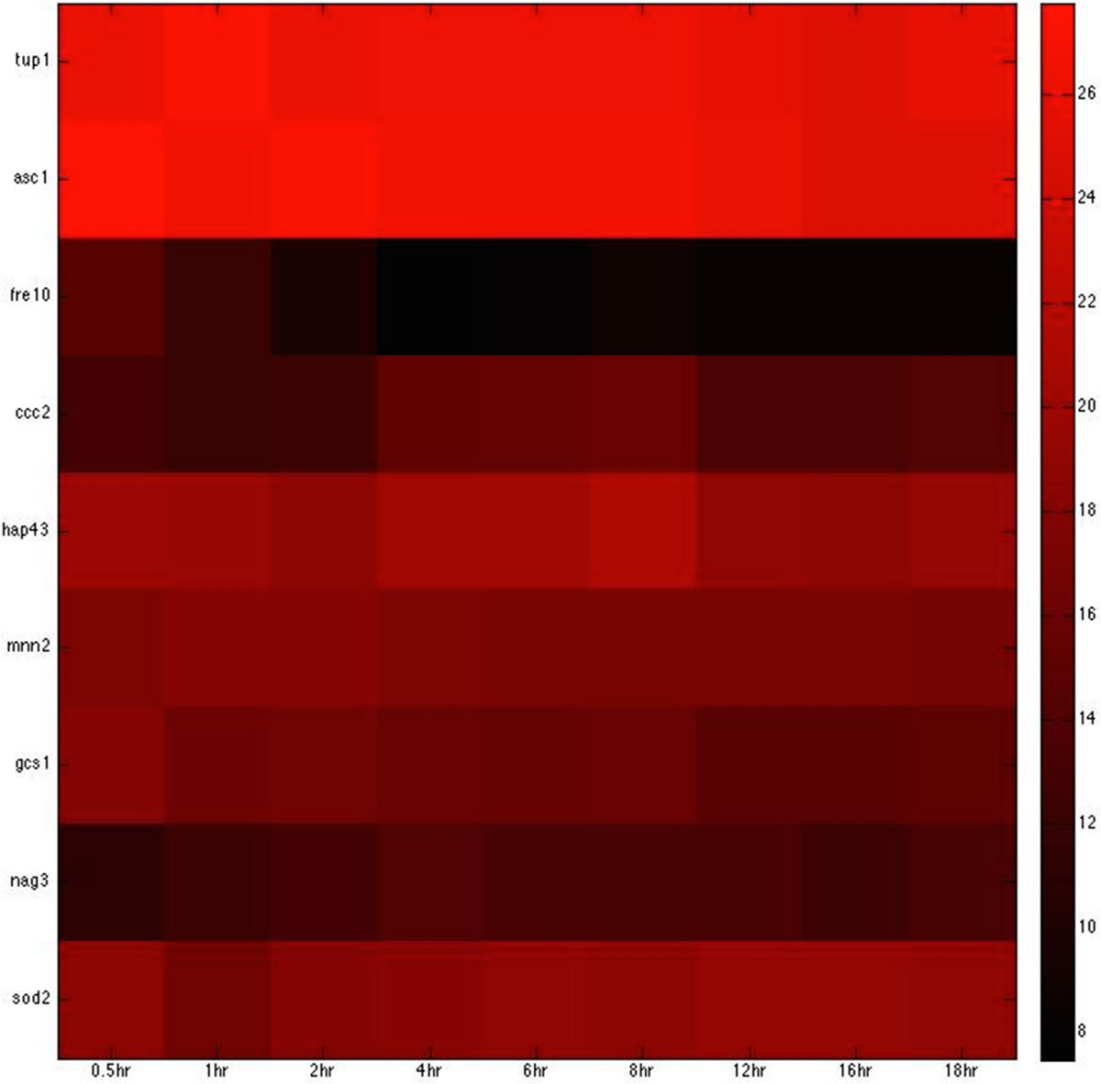
**Heatmap of *C*. albicans iron-related virulence proteins with statistically significant interactions in the adhesion stage.** The heatmap of the listed proteins in table 2 shows the expression levels of the time-course microarray data with nine times.

**Figure 3:**
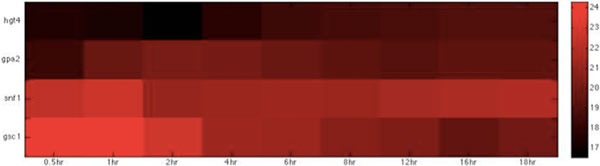
**Heatmap of *C*. albicans glucose-related virulence proteins with statistically significant interactions in the adhesion stage.** The heatmap of the listed proteins in table 3 shows the expression levels of the time-course microarray data with nine times.

**Figure 4:**
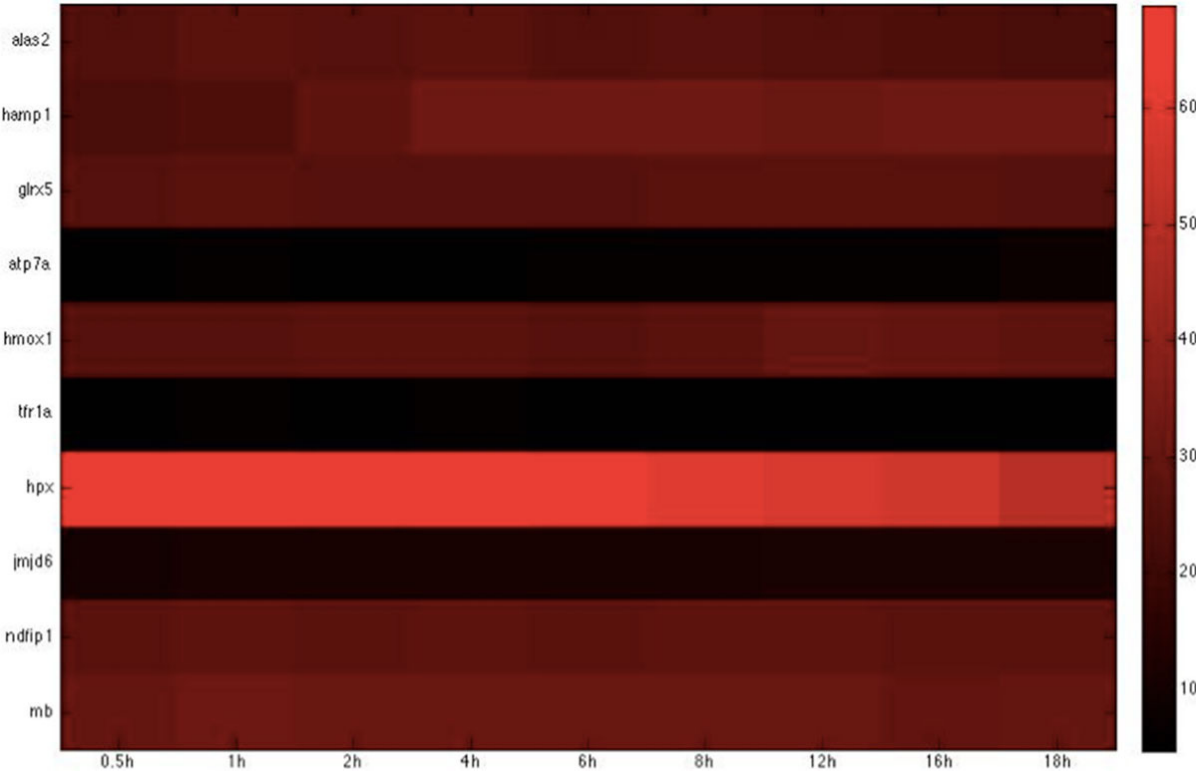
**Heatmap of Zebrafish iron-related immune proteins with statistically significant interactions in the adhesion stage**. The heatmap of the listed proteins in table 4 shows the expression levels of the time-course microarray data with nine times.

**Figure 5:**
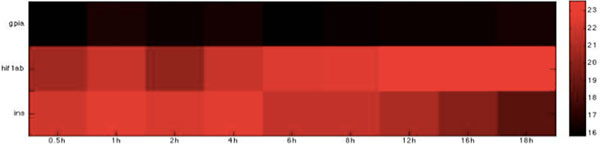
**Heatmap of Zebrafish glucose-related immune proteins with statistically significant interactions in the adhesion stage**. The heatmap of the listed proteins in table 5 shows the expression levels of the time-course microarray data with nine times.

**Figure 6:**
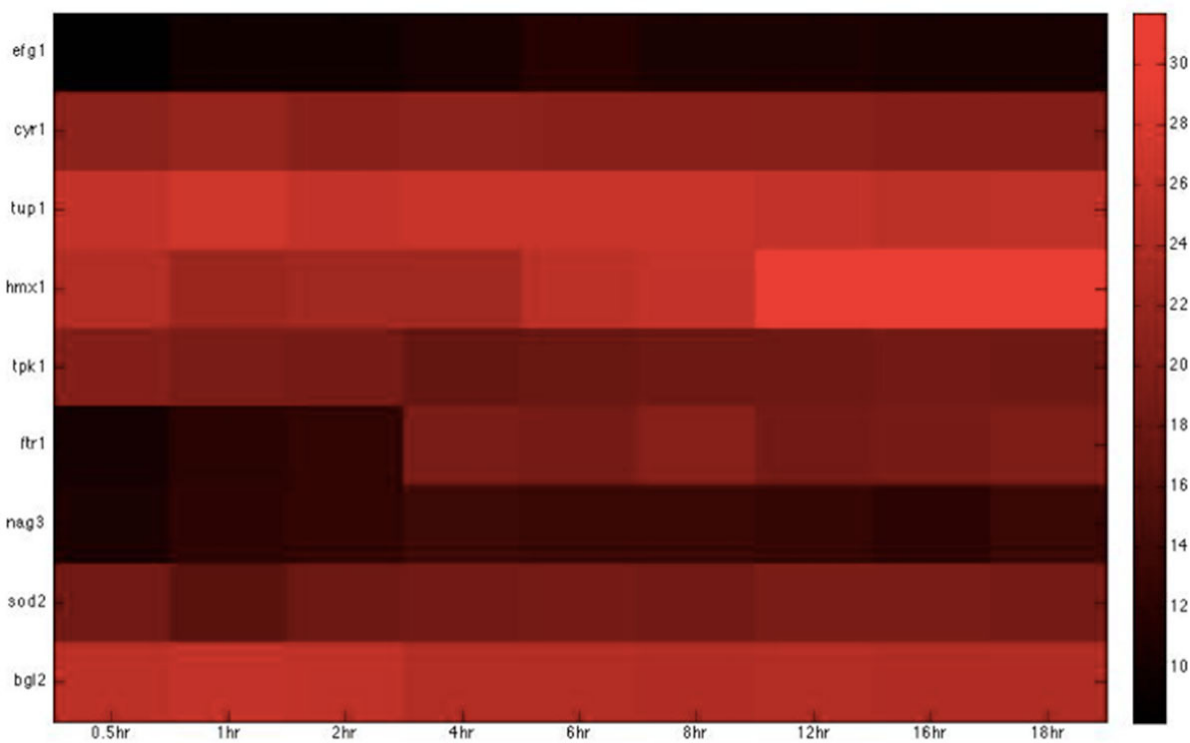
**Heatmap of *C*. albicans iron-related virulence proteins with statistically significant interactions in the invasion stage.** The heatmap of the listed proteins in table 6 shows the expression levels of the time-course microarray data with nine times.

**Figure 7:**
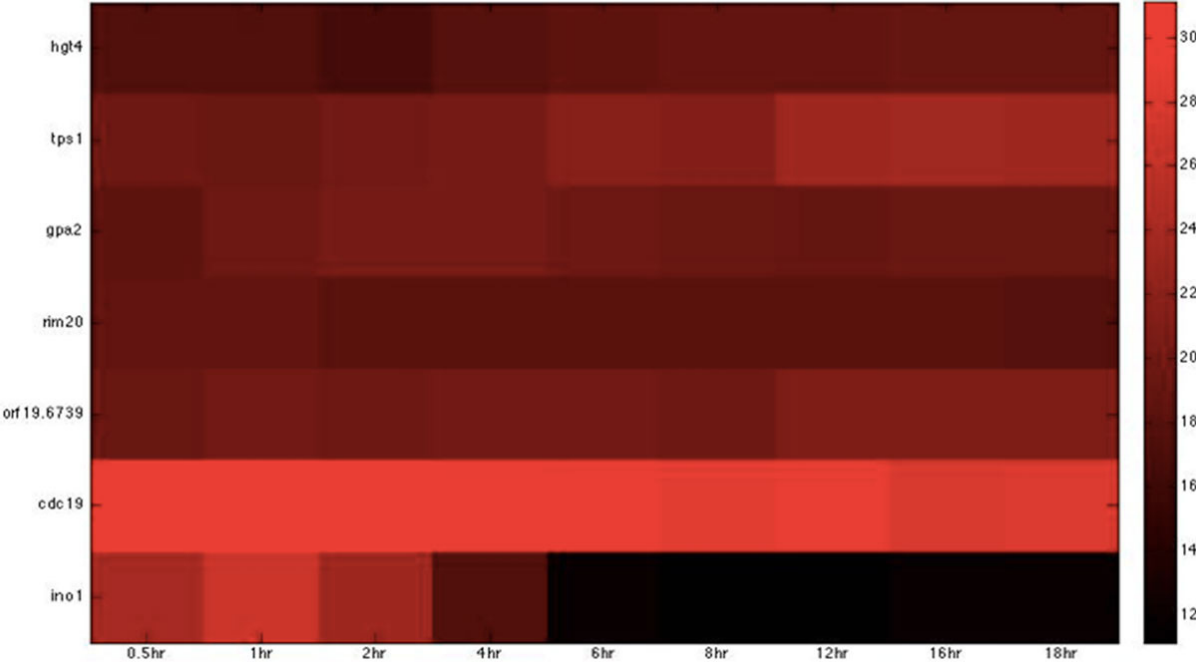
**Heatmap of *C*. albicans glucose-related virulence proteins with statistically significant interactions in the invasion stage. **The heatmap of the listed proteins in table 7 shows the expression levels of the time-course microarray data with nine times.

**Figure 8:**
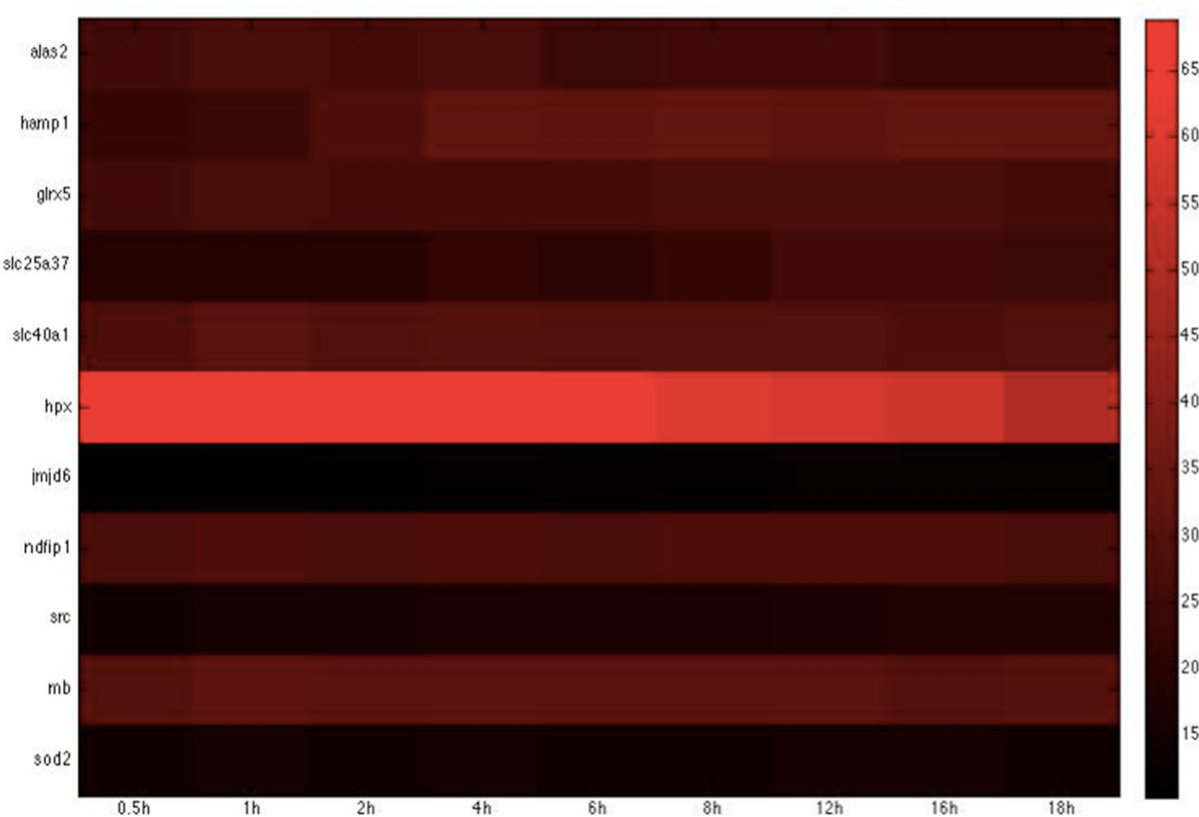
**Heatmap of Zebrafish iron-related immune proteins with statistically significant interactions in the invasion stage**. The heatmap of the listed proteins in table 8 shows the expression levels of the time-course microarray data with nine times.

**Figure 9:**
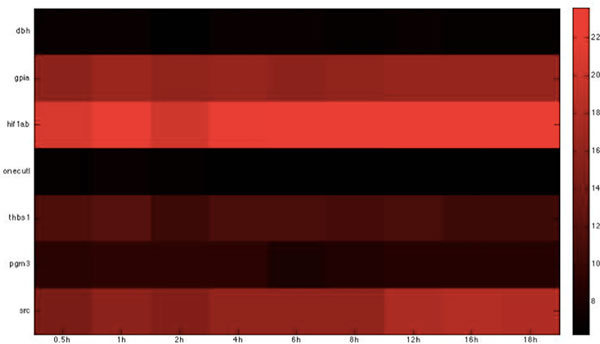
**Heatmap of Zebrafish glucose-related immune proteins with statistically significant interactions in the invasion stage**. The heatmap of the listed proteins in table 9 shows the expression levels of the time-course microarray data with nine times.

**Figure 10:**
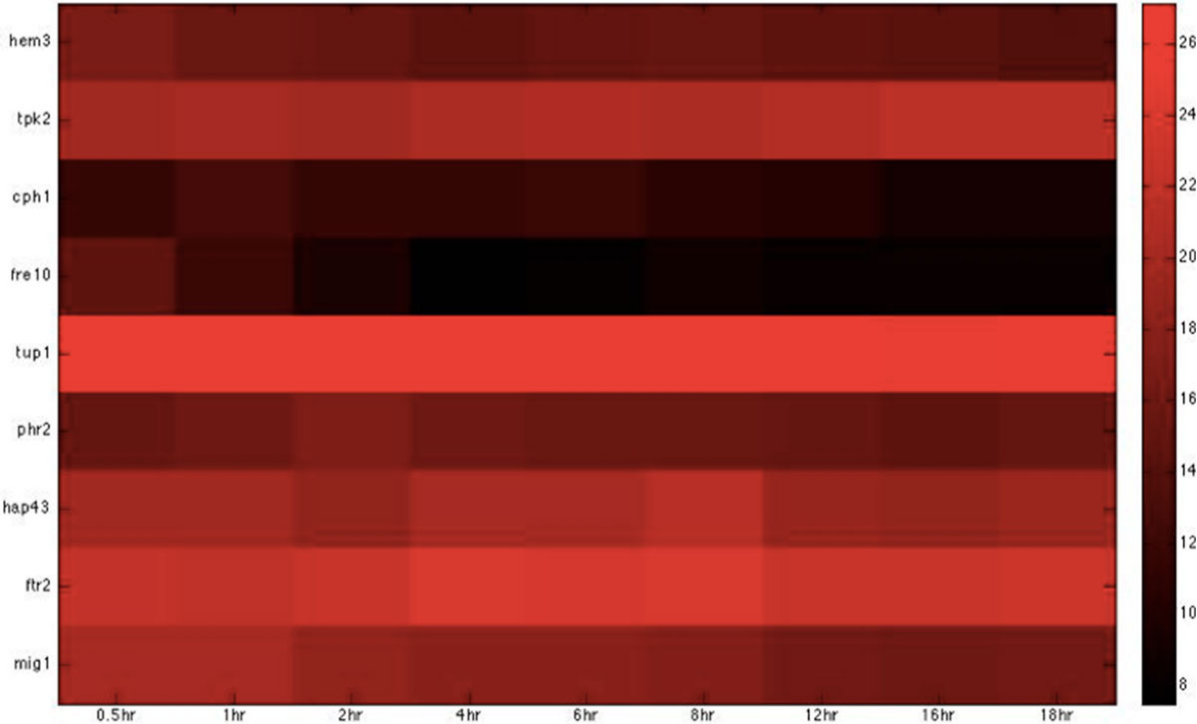
**Heatmap of *C*. albicans iron-related virulence proteins with statistically significant intercations in the damage stage.** The heatmap of the listed proteins in table 10 shows the expression levels of the time-course microarray data with nine times.

**Figure 11:**
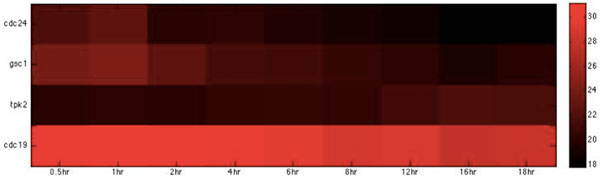
**Heatmap of *C***. albicans glucose-related virulence proteins with statistically significant interactions in the damage stage. The heatmap of the listed proteins in table 11 shows the expression levels of the time-course microarray data with nine times.

**Figure 12:**
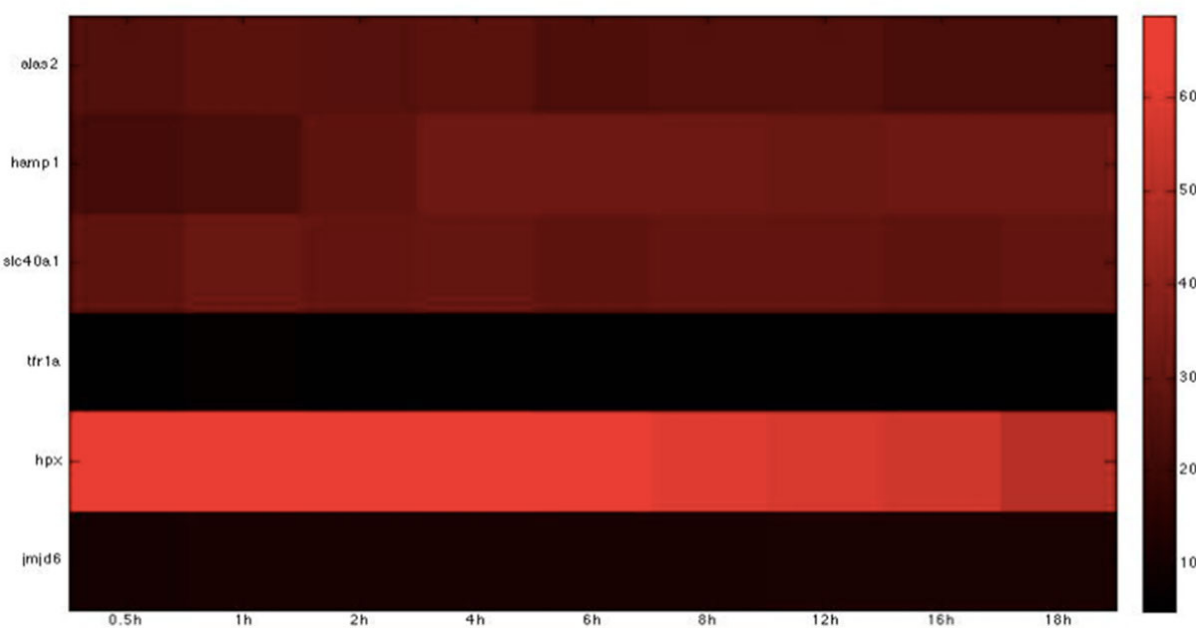
**Heatmap of Zebrafish iron-related immune proteins with statistically significant interactions in the damage stage**. The heatmap of the listed proteins in table 12 shows the expression levels of the time-course microarray data with nine times.

Based on the host-pathogen interaction network constructed above, we were able to obtain the number of linkages of each target protein in zebrafish and the corresponding interspecies interaction proteins in *C. albicans *during the three infection stages, identifying important hub proteins in both species (see Additional file 1.xlsx in supplementary file for details). To further identify the target proteins and interspecies interaction proteins that had statistically significant roles during each of the three infection stages, we randomized our simultaneously quantified time-course microarray data with respect to time-points. It should be noted that since zebrafish proteins were set as targets for interspecies interaction, this randomization was only applied to zebrafish and not to *C. albicans *data. Microarray data of zebrafish target immune proteins in the iron-related and glucose-related pool were each randomized 10,000 times. Interspecies interaction networks were constructed and the number of linkages of each protein recorded in each run, yielding the distribution of the number of linkages for each protein in the three infection stages. A protein was determined to be statistically significant in the corresponding stage if its original number of linkages was located closer to the low-probability area rather than the mean at a significance level of p < 0.05 with Student's t-test. Zebrafish target proteins or *C. albicans *interspecies interaction proteins were determined to be statistically significant for each of the three infection stages (see Table 2, 3, 4, 5, 6, 7, 8, 9, 10, 11, 12, 13 for the complete lists and Figure 2, 3, 4, 5, 6, 7, 8, 9, 10, 11, 12, 13 for the corresponding heatmap. See Additional file 2.zip for the original heatmap).

**Table 2:** *C.* albicans iron-related virulence proteins with statistically significant interactions in the adhesion stage.

iron-related virulence protein	p-value	GO annotation
*tup1*	0.023	cell-cell adhesion/reductase activity

*asc1*	0.024125	cell adhesion

*fre10*	0.0035	reductase activity

*ccc2*	0.02575	copper ion transport

*hap43*	0.0325	iron assimilation

*mnn2*	0.023	high-affinity iron ion transport

*gcs1*	0.03475	

*nag3*	0.01025	

*sod2*	0.043	

The list of proteins with statistically significant interactions. Their corresponding *p*-values and GO annotations are also listed.

**Table 3:** *C.* albicans glucose-related virulence proteins with statistically significant interactions in the adhesion stage.

glucose-related virulence protein	p-value	GO annotation
*hgt4*	0.0013	glucose sensor

*gpa2*	0.0022	sensing of glucose

*snf1*	0.0056	cell adhesion

*gsc1*	0.042	

The list of proteins with statistically significant interactions. Their corresponding *p*-values and GO annotations are also listed.

**Table 4:** Zebrafish iron-related immune proteins with statistically significant interactions in the adhesion stage.

Zebrafish iron-related immune protein	p-value	GO annotation
***alas2***	0.0191	heme biosynthetic process

***hamp1***	0.0253	defense response to bacterium

***glrx5***	0.0221	electron carrier activity

***atp7a***	0.0158	copper ion transport

***hmox1***	0.0154	iron ion homeostasis

***tfr1a***	0.0251	biosynthesis of hemoglobin

***hpx***	0.003	heme transport

***jmjd6***	0.0189	macrophage activation

***ndfip1***	0.0142	iron homeostasis

***mb***	0.0008	

The list of proteins with statistically significant interactions. Their corresponding *p*-values and GO annotations are also listed.

**Table 5:** Zebrafish glucose-related immune proteins with statistically significant interactions in the adhesion stage.

Zebrafish glucose-related immune protein	p-value	GO annotation
***gpia***	0.004833	humoral immune response

***hif1ab***	0.0065	inflammatory response

***ins***	0.006833	glucose transport/T cell activation

The list of proteins with statistically significant interactions. Their corresponding *p*-values and GO annotations are also listed.

**Table 6:** *C.* albicans iron-related virulence proteins with statistically significant interactions in the invasion stage.

iron-related virulence protein	p-value	GO annotation
*efg1*	0.04328	hyphal formation and filamentous growth

*cyr1*	0.024	hyphae formation

*tup1*	0.00002456	negative regulator of filamentous growth

*hmx1*	0.03085	iron ion homeostasis

*tpk1*	0.029857143	hyphae formation

*ftr1*	0.028571429	high-iron affinity permease

*nag3*	0.000142	

*sod2*	0.029	

*bgl2*	0.04	

The list of proteins with statistically significant interactions. Their corresponding *p*-values and GO annotations are also listed.

**Table 7:** *C. *albicans glucose-related virulence proteins with statistically significant interactions in the invasion stage.

glucose-related virulence protein	p-value	GO annotation
*hgt4*	0.0034	glucose transportation activity/virulence

*tps1*	0.011	hyphal formation

*gpa2*	0.027	hyphal formation

*rim20*	0.003	morphological transition

*orf19.6739*	0.04	

*cdc19*	0.03267	

*ino1*	0.017	

The list of proteins with statistically significant interactions. Their corresponding *p*-values and GO annotations are also listed.

**Table 8:** Zebrafish iron-related immune proteins with statistically significant interactions in the invasion stage.

Zebrafish iron-related immune protein	p-value	GO annotation
*alas2*	0.028833	heme biosynthetic process

*hamp1*	0.0325	defense response to bacterium

*glrx5*	0.02267	electron carrier activity

*slc25a37*	0.028167	hemopoiesis

*slc40a1*	0.02667	hemoglobin biosynthesis process

*hpx*	0.0325	heme transport

*jmjd6*	0.0265	macrophage activation

*ndfip1*	0.0225	iron homeostasis

*src*	0.027667	

*mb*	0.025167	

*sod2*	0.0205	

The list of proteins with statistically significant interactions. Their corresponding *p*-values and GO annotations are also listed.

**Table 9:** Zebrafish glucose-related immune proteins with statistically significant interactions in the invasion stage.

Zebrafish glucose-related immune protein	p-value	GO annotation
*dbh*	0.012167	immunity
*gpia*	0.0155	humoralimuune response

*hif1ab*	0.0000667	inflammatory response

*onecutl*	0.0145	B cell differentiation

*thbs1*	0.013667	immune response

*pgm3*	0.013167	

*src*	0.030667	

A) The list of proteins with statistically significant interactions. Their corresponding *p*-values and GO annotations are also listed.

**Table 10:** *C.* albicans iron-related virulence proteins with statistically significant intercations in the damage stage.

iron-related virulence protein	p-value	GO annotation
*hem3*	0.0202	pathogenesis
*tpk2*	0.0273	virulence and filamentous growth

*cph1*	0.0488	filamentous growth and pathogenesis

*fre10*	0.0021	iron ion transport

*tup1*	0.04285	hyphae formation

*phr2*	0.03345	pathogenesis

*hap43*	0.0137	virulence

*ftr2*	0.047	iron ion transporter activity

*mig1*	0.04685	

The list of proteins with statistically significant interactions. Their corresponding *p*-values and GO annotations are also listed.

**Table 11:** *C.* albicans glucose-related virulence proteins with statistically significant interactions in the damage stage

glucose-related virulence protein	p-value	GO annotation
*cdc24*	0.0023	hyphal growth/ pathogenicity

*gsc1*	0.017	virulence

*tpk2*	0.003	virulence

*cdc19*	0.037	

The list of proteins with statistically significant interactions. Their corresponding *p*-values and GO annotations are also listed.

**Table 12:** Zebrafish iron-related immune proteins with statistically significant interactions in the damage stage.

Zebrafish iron-related immune protein	p-value	GO annotation
*alas2*	0.01225	heme biosynthesis

*hamp1*	0.01825	defense response to bacterium

*slc40a1*	0.0125	hemoglobin biosynthesis process

*tfr1a*	0.01925	biosynthesis of hemoglobin

*hpx*	0.02025	heme transport

*jmjd6*	0.01975	macrophage activation

The list of proteins with statistically significant interactions. Their corresponding *p*-values and GO annotations are also listed.

**Table 13:** Zebrafish glucose-related immune proteins with statistically significant interactions in the damage stage.

Zebrafish glucose-related immune protein	p-value	GO annotation
*edn1*	0.022833	glucose transport

*ptenb*	0.003833	

The list of proteins with statistically significant interactions. Their corresponding *p*-values and GO annotations are also listed.

**Figure 13:**
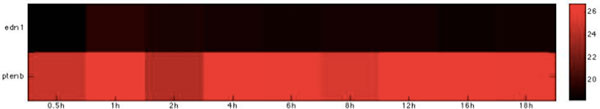
**Zebrafish glucose-related immune proteins with statistically significant interactions in the damage stage**. The heatmap of the listed proteins in table 13 shows the expression levels of the time-course microarray data with nine times.

### Estimation of robustness of interspecies interaction networks

The estimation of robustness follows from that in [9] and is described briefly below. After constructing the dynamic model of the interspecies protein interaction network, we estimate the network robust based the discrete-time dynamic model. Assume that there are *z *proteins in an interaction network, we can use the following linear discrete-time dynamic model to approximate the interaction network

$$X\left[t+1\right]=AX\left[t\right]+H+n\left[t\right]$$

$A=\left[\begin{array}{ccccc} \alpha_{1,1} & \alpha_{1,2} & \cdots \; & \cdots \; & \alpha_{1,Z}\\ \alpha_{2,1} & \ddots & & & \\ \vdots & & \alpha_{ij} & & \\ \vdots & & & \ddots & \\ \alpha_{Z,1} & & & & \alpha_{Z,Z} \end{array}\right]$, $H=\left[\begin{array}{c} h_{1}\\ h_{2}\\ \vdots \\ \vdots \\ h_{Z} \end{array}\right]$ (7)

where X[t] = [ x1[t] x2[t] ... xz[t] ]^T ^represents the protein activity level with a total of z proteins in the network. And the interaction matrix A represents the interaction of protein with interaction ability α_ij _between protein i and protein j. H is the vector of the basal level of each protein. When the system reaches steady state X_S_, the discrete-time dynamic model (7) could be rewritten as

*X_S _= AX_S _+ H*, or *X_S _= (I-A)^-1^H *(8)

Next, we shift the dynamic system by X_S _(i.e., X[t] = X'[t] + X_S _). Then, we can subtract (7) from (8) to obtain the shifted dynamic system as follow:

(9) $$X^{\prime }\left[t+1\right]=AX^{\prime }\left[t\right]$$

Now we can begin to measure the robustness at the new origin where ${\text{H}}^{\prime }\left[\text{t}\right]=0$. When a system suffers from intrinsic perturbations such as thermal fluctuation, gene mutation or any other process noise, such perturbation is represented by adding an additional perturbation ΔA=ηA in to system (9) to obtain:

(10) $$X^{\prime }\left[t+1\right]=A\left(1+\eta \right)X^{\prime }\left[t\right]$$

Higher η value means that the system suffers greater intrinsic perturbation which would affect the stability of the dynamic system. If a system can tolerate larger η then we can say that system is more robust. Therefore we define the maximum η value, $\eta^{\circ }$ in (10) such that the system is still stable as the "robustness" of the network [9]. According to quadratic stability theory [19,20], if the perturbative network described in (10) is robustly stable, the following inequality has a positive definite solution P=P^T^>0:

(11) $$\left[A\left(1+\eta \right)\right]TP\left[A\left(1+\eta \right)\right]\leq P$$

Since robustness is the intrinsic perturbation tolerance ability with respect to the maintenance of steady state, the system must be stable and (11) should be satisfied. Therefore, the robustness value $\eta^{\circ }$ is found by increasing η in (11) such that the inequality in (11) can be satisfied with a positive definite solution P=P^T^>0 until reaching the maximum$\eta^{\circ }$.

## Results

In this study, we aimed to estimate the robustness for three distinctive infection stages (adhesion, invasion, and damage stage) from iron and glucose interspecies protein interaction networks. Based on simultaneously quantified time-course microarray gene expression data and the procedure described in Figure 1, iron and glucose interspecies protein interaction networks for three infection stages were constructed (see Additional file 3.zip in supplementary file for the complete description of the constructed interspecies networks).

**Figure 14:**
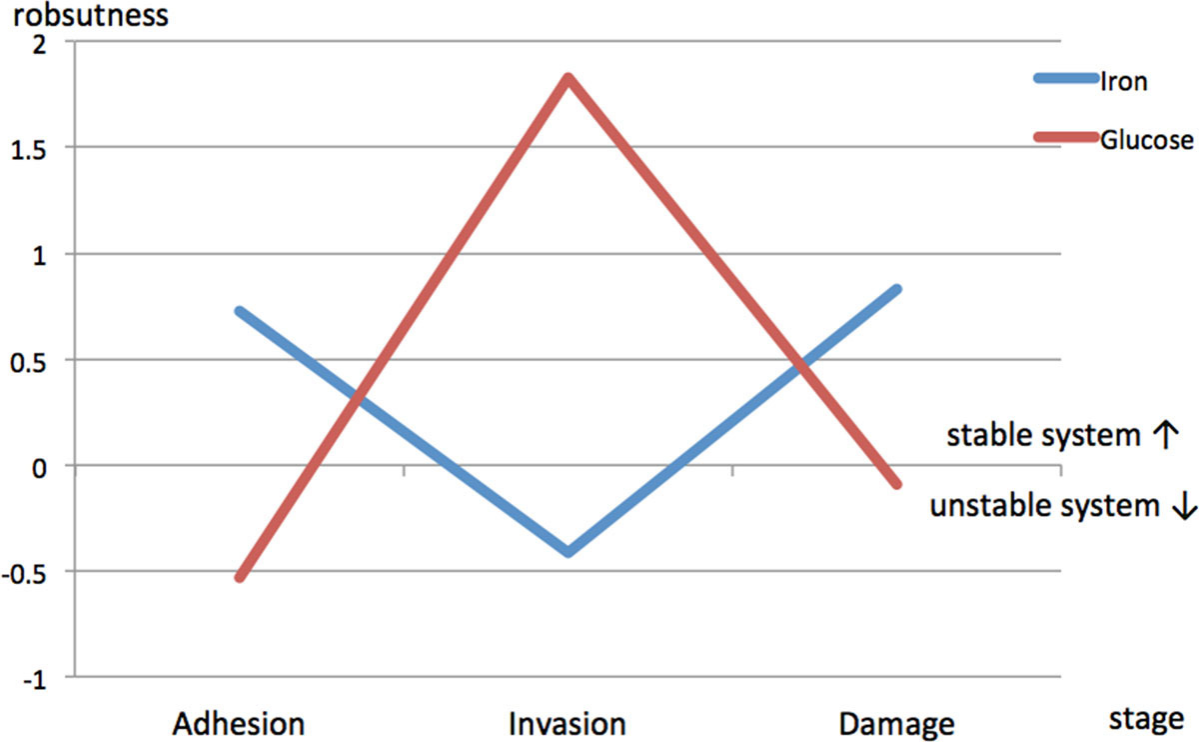
**The robustness of the iron and glucose competition interspecies network at each infection stage**. A negative value of robustness represents an unstable system, and a positive value represents tolerance of intrinsic perturbation. In the adhesion stage, iron is the focus resource. In the invasion stage, glucose becomes important due to hyphal formation. In the damage stage, iron competition becomes stable for transportation and storage but glucose competition gets out of control for damaged hosts.

In the adhesion stage, we identified 65 PPIs in zebrafish and 273 interspecies interactions between *C. albicans *and zebrafish in the iron host-pathogen interspecies interaction network with robustness $\eta^{\circ }$ equal to 0.7274 (Table 14, Figure 14), A negative value of robustness represents an unstable system, and a positive value provides a quantitative measure about how much the systems can endure the intrinsic perturbation. We identified 99 PPIs and 168 interspecies interactions in the glucose network with robustness $\eta^{\circ }$ equal to -0.6306. In the invasion stage, we identified 96 PPIs and 271 interspecies interactions in the iron network with robustness $\eta^{\circ }$ equal to -0.4131 and 76 PPIs and 294 interspecies interactions in the glucose network with robustness $\eta^{\circ }$ equal to 1.8281. In the damage stage, we identified 78 PPIs and 204 interspecies interactions in the iron network and 69 PPIs and 286 interspecies interactions in the glucose network with robustness $\eta^{\circ }$ equal to 0.8301 and -0.088.

**Table 14:** The value of the robustness for the iron and glucose interspecies interaction networks at each infection stage.

Stage	Adhesion	Invasion	Damage
Iron	0.7274	-0.4131	0.8301

Glucose	-0.5306	1.8281	-0.088

Based on the constructed networks, we further identified proteins with statistically significant interactions (p < 0.05) for three distinct infection stages. In the adhesion stage, we identified nine significantly interactive iron-related virulence proteins and four glucose-related virulence proteins in *C. albicans*, and 10 significantly interactive iron-related immune proteins and three glucose-related immune proteins in zebrafish; in the invasion stage, nine iron-related and nine glucose-related virulence proteins, and 11 iron-related and seven glucose-related immune proteins; and in the damage stage, nine iron-related and four glucose-related virulence proteins, and six iron-related and two glucose-related immune proteins.

We additionally investigated the biological processes and molecular functions of these proteins by data mining the existing literature. Based on literature findings, proteins were further divided into three main categories for each infection stage: significantly interactive proteins with strong evidence, significantly interactive proteins with partial evidence, and those that were not yet known to be involved in the infection process.

The proteins with strong evidence are well known to have direct relationships with the designated infection stage between *C. albicans *and zebrafish. The results from this category confirm the reliability of our method and validate our proposed model. More details for proteins with strong evidence are listed in the Additional file 4.docx in the supplementary file.

The identified proteins with partial evidence represent proteins that were identified to only have indirect connections with the interactions in the designated infection stage. Proteins from this group can be viewed as predictions from our constructed networks since they may be essential for the host-pathogen interaction during infection for the corresponding infection stage. Furthermore, this group may provide new insights into how the infective process progresses within both the host and pathogen. The unknown group includes proteins that have not yet been identified as influential on infection due to of previously associated findings, but may still play a crucial role in resource competition in host-pathogen interactions.

### Adhesion stage

After investigating the host-pathogen interspecies interaction network in the adhesion stage, we identified 273 and 168 interspecies interactions between *C. albicans *and zebrafish in iron and glucose competition, respectively.

For the iron competition interspecies network, its robustness was estimated to be 0.7274, which was far lager than the robustness estimated in the glucose competition interspecies network (-0.5306). The robustness value of glucose competition interspecies network is negative since this dynamic system is unstable (or not functional) during the adhesion stage. In other words, this indicates that glucose competition is not essential between *C. albicans *and zebrafish during the adhesion stage. On the other hand, positive robustness of the iron competition interspecies network indicates that iron competition is active and that the zebrafish iron competition network can resist intrinsic perturbation with a ratio of 0.7274. Compared with the glucose competition interspecies network, we can infer that iron competition is the main focus between *C. albicans *and zebrafish in the adhesion stage.

We further identified proteins with statistically significant interactions in the adhesion stage, including nine virulence iron-related proteins (GO annotation: adhesion activity - 22.2%, metal ion transport - 11.1%, reductase activity - 11.1%, cellular iron ion homeostasis - 22.2%) and four virulence glucose-related proteins (GO annotation: glucose sensor - 50%, cell adhesion - 25%) in *C. albicans *and 10 immune iron-related proteins (GO annotation: hematopoiesis-related process - 50%, immune response - 20%, metal ion transport - 20%) and three immune glucose-related proteins (GO annotation: immune response - 66.7%, glucose transport - 33.3%) in zebrafish by their corresponding *p*-values (Table 2, 3, 4, 5, Figure 2, 3, 4, 5).

### Differentially regulated proteins with partial evidence

For the *C. albicans *iron host-pathogen interspecies interaction network, *fre10, tup1, ccc2*, and *hap43 *were identified in the adhesion stage.

*Fre10 *is reported to be responsible for reductase activity and the encoding of cell surface ferric reductase [21]. Iron uptake from transferrin is reported to be impaired in *fre10 *mutants [21]. The identification of *fre10 *in the adhesion stage suggests that *C. albicans *is also beginning to activate the associated mechanisms necessary to compete for iron resources in the host at this point. *Tup1 *is a global regulator of morphology and metabolism. It plays a key role not only in iron regulation of ferric reductase and ferrous transport activities, but also in iron-dependent gene regulation [22]. The identification of *tup1 *suggests that most of the iron-related genes are active in the adhesion stage in order to acquire iron from the host. *Hap43 *has been shown to be an essential protein for the growth of *C. albicans *in low-iron environments and also for *C. albicans *virulence in a mouse model [23]. It is also involved in iron assimilation [24]. The identification of *hap43 *in the early adhesion stage indicates that *C. albicans *needs to absorb as much iron as possible at this point, since the iron concentration is relatively low during this initial stage of the infection. *Ccc2 *also plays a pivotal role in iron acquisition during the adhesion stage. It has been reported that *ccc2 *may be involved in iron reductase activity [25]. The process for the transition between ferrous and ferric iron often involves the donation and acquisition of electrons, which may cause free radical toxicity inside the cell. To prevent the production of toxic free radicals, ferrous iron is oxidized to ferric iron by multicopper oxidase activity [26]. *Mnn2 *has been shown to be responsible for high-affinity iron ion transport [27]. Since iron is highly important for the pathogen to continue attacking the host, the related transportation mechanisms must also be activated.

For iron competition in zebrafish during this early adhesion stage, we identified *atp7a, glrx5, hmox1, tfr1a*, and *hpx *in the iron host-pathogen interaction network. *atp7a *has been shown to be involved in copper ion transport [28]. Since *ccc2*, which is involved in preventing damage by toxic free radicals via its reductase activities [29], was identified in *C. albicans*, the identification of *atp7a *in zebrafish implies that the host also invokes a similar mechanism to prevent the damage caused by the intracellular toxicity of free radicals. The presence of *glrx5*, a member of the glutaredoxin family, suggests that this family is involved in electron carrier activities [30]. The identification of *glrx5 *is not surprising, as reductase activity is present in both the adhesion and invasion stage and the process depends on the mobilization of electrons. *Hmox1 *is reported to redirect heme iron to the extracellular region, thereby lowering the accumulation of intracellular iron [31]. Its identification may therefore explain how *C. albicans *exploits the iron resource from zebrafish, since the acquisition of extracellular iron is easier than that of intracellular iron. It has also been shown that *ndfip1*-deficient mice have impaired DMT1 regulation and iron homeostasis [32]. The identification of *ndfip1 *implies that control of the intracellular iron concentration is also important in the competition for iron. *tfr1a*, an erythroid-specific isoform of transferrin receptor 1 encoded by *chianti *(*cia*), is reported to be essential for iron acquisition by erythrocytes [33]. Its presence implies that this protein may be responsible for iron homeostasis in zebrafish during infection by maintaining the iron concentration. *Hpx *is also widely reported to be related to heme transport and cellular iron ion homeostasis [34]. In this early adhesion stage, the immune response in the zebrafish must be activated to fight the invasion of *C. albicans*. The protein *jmjd6 *has been shown to be involved in the phagocytosis of apoptotic cells and associated immune functions [35]. The identification of this immune-related protein in the adhesion stage from our interspecies network suggests that zebrafish also activate their immune functions to target the pathogen at this stage.

For glucose competition, *gpia, hif1ab*, and *ins *were identified. *gpia *is known to be involved in the humoral immune response [36], while *hif1ab *is reported to be a critical link between inflammation and oncogenesis and is responsive to interleukin-1 [37]. The identification of *gpia *and *hif1ab *implies that the host has activated its immune functions to eliminate the pathogen during the early adhesion stage.

*ins *stimulates glucose transport by the nitric oxide/cyclic GMP pathway and plays a pivotal role in the uptake of glucose [38]. It is also related to T cell activation [39]. Its identification suggests that glucose transportation is also crucial for efficient uptake of glucose by zebrafish and that zebrafish activate T cells for defensive mechanisms in the early adhesion stage. We observed that most of these identified glucose-related proteins during the adhesion stage were related to biological processes associated with immunity. This interesting observation suggests that the uptake of glucose may support immune functions.

**Figure 15:**
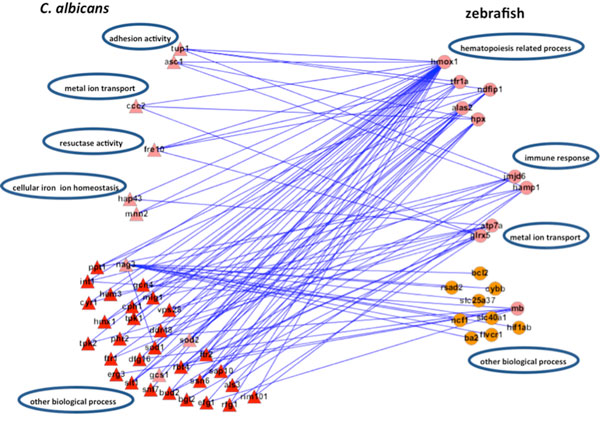
**The sub-network for the iron host-pathogen interspecies interaction network in the adhesion stage**. There were 273 interspecies interactions between *C. albicans *and zebrafish in the iron host-pathogen interspecies interaction network in the adhesion stage. There were nine iron-related virulence proteins identified as significantly interactive for *C. albicans *(pink triangles) and 10 iron-related immune proteins for zebrafish (pink circles). The major functional modules for *C. albicans *in this early infection stage were identified as adhesion activity (*tup1, asc1*), metal ion transport (*ccc2*), reductase activity (*fre10*), and cellular iron ion homeostasis (*hap43, mnn2*), while the main activities for zebrafish were identified as the immune response (*jmjd6, hamp1*), metal ion transport (*atp7a, glrx5*), and hematopoiesis-related processes (*hmox1, tfr1a, ndfip1, alas2, hpx*).

These constructed interspecies interactions of iron and glucose networks provided some insights into how the host-pathogen interaction proceeds during this early stage of infection. Based on the resulting networks (Figure 15), the main goal of the pathogen in the adhesion stage is to adhere to the host and to begin to detect iron and glucose surrounding the attachment site. At the same time, the host activates the immune response to defend against the pathogen, and the biological processes necessary for the production of essential resources are also implemented. In addition, the biological process of metal ion transport is simultaneously activated in both host and pathogen. This suggests that free ions and associated nutrients begin to flow between zebrafish and *C. albicans *due to the latter's adherence. Following this metal ion transport process, cellular iron ion homeostasis in *C. albicans *and the hematopoiesis-related process in zebrafish are also activated to maintain the concentration of cellular free ions.

### Invasion stage

In our constructed host-pathogen interspecies interaction network, we identified 271 and 294 interspecies interactions between *C. albicans *and zebrafish in the iron and glucose competition, respectively.

For the iron competition interspecies network, its robustness was estimated to be -0.4131, which was far smaller than the robustness estimated in the glucose competition interspecies network (1.8281). We observed that robustness in the iron competition interspecies network changes from 0.7274 to -0.4131 in the adhesion and invasion stage, respectively. This indicates that iron competition is not the main focus during the invasion stage. On the other hand, positive robustness of the glucose competition interspecies network indicates that glucose competition is active and that the zebrafish glucose competition network can resist intrinsic perturbation with a ratio of 1.8281 between *C. albicans *and zebrafish and during the invasion stage. Compared with the iron competition network, we can infer that glucose competition is the main focus between *C. albicans *and zebrafish in the invasion stage.

We further identified proteins of statistically significant interactivity in *C. albicans *during the invasion stage, comprising nine virulence iron-related proteins (GO annotation: hyphal formation - 22.2%, filamentous growth - 22.2%, cellular iron ion homeostasis - 22.2%) and seven virulence glucose-related proteins (GO annotation: hyphal formation - 28.6%, glucose transportation - 14.2%, morphological transition - 14.2%). Among these proteins, most were related to filamentous development and hyphae formation. This is not surprising given that non-filamentous *C. albicans *mutants are avirulent [40]. In zebrafish, we identified 11 iron-related immune proteins (GO annotation: hematopoiesis-related process - 45.5%, metal ion transport - 9%, immune response - 18.1%) and seven glucose-related immune proteins (GO annotation: immune response - 71.4%) as significant (Table 6, 7, 8, 9, Figure 6, 7, 8, 9).

### Differentially regulated proteins with partial evidence

In *C. albicans*, we identified *hmx1, tup1, tpk1, and ftr1 *from the iron host-pathogen interspecies interaction network in the invasion stage. *Hmx1 *is related to cellular iron ion homeostasis. In the hemoglobin uptake and degradation system, *hmx1 *encodes an active hemoxygenase that degrades hemoglobin to  α-biliverdin [41]. In our network, *hmx1 *was identified as being most active in the invasion stage, indicating that hemoglobin degradation occurs mainly during the invasion stage. Moreover, *hmx1 *is positively regulated by iron deprivation [42]. This could imply that *C. albicans *acquires iron from iron-binding proteins within the host during the adhesion stage. The main resource that *C. albicans *competes for upon entering the host is glucose, which enhances hyphae formation. As a result, proteins involved in maintaining the concentration of cellular iron ions are necessary for the invasion stage, which explains the identification of *hmx1 *in network at this stage. *tup1 *is known to be a negative regulator of filamentous growth [43]. This is interesting given that *C. albicans *was thought to initiate hyphae formation and filamentous growth during the invasion stage to enter the host organism. Note that the host-pathogen interaction network we constructed is a dynamically evolving system. Every protein may potentially play two or more roles in the dynamic system, suggesting that *tup1 *may play roles other than the one discussed above.

*tpk1 *was shown to be involved in activating downstream transcriptional regulators, including the global regulator *efg1*. Interestingly, *efg1 *was also identified in our network during the invasion stage. This suggests that the repression of the protein kinase A subunit *tpk1 *activates *efg1 *to support hyphae formation [44]. *Ftr1 *is a high-iron affinity permease that is involved in iron ion transport. Its deletion prevents systemic infection in mice, and mutant fungi exhibit severe growth defects in low-iron medium [45].

The gene *rim20 *was identified as involved in glucose competition. Indeed, several findings indicate that the *rim101 *transduction pathway regulates *C. albicans *virulence [46,47]. The identification of *rim20 *in our glucose host-pathogen interspecies interaction network in the invasion stage thus indicates that *rim20 *is pivotal in morphological transition and hyphal formation. Further experimental studies should be performed to verify its specific role in infection.

In zebrafish, we identified *slc25a37, slc40a1*, and *ndfip1 *from the iron host-pathogen interspecies interaction network in the invasion stage.

*slc25a37 *is reported to be associated with hemopoiesis [48]. The appearance of this protein in the invasion stage may suggest that, due to the adherence and invasion of *C. albicans*, the host requires more iron and oxygen for hyperemia at the attachment site. *Slc40a1 *is important in the hemoglobin biosynthesis process [49,50]. The identification of both *slc25a37 *and *slc40a1 *implies that the host counters the loss of iron resources due to pathogen invasion by producing more iron. In the invasion stage, we can further infer that *C. albicans *begins the utilization and transportation of iron while the zebrafish activates relevant heme biosynthesis and red blood cell-producing processes to maintain the balance of all essential cellular elements.

Focusing on glucose-related proteins, *dbh, gpia, hif1ab, onecutl*, and *thbs1 *were identified. It has been shown that a deficiency in dopamine beta-hydroxylase (*dbh*) leads to impaired cellular immunity [51]. The identification of this protein may suggest that immune-related functions are continuously implemented by zebrafish to combat the pathogen in the invasion stage. *Onecutl *is known to be involved in B cell differentiation [52]. Furthermore, hepatocyte nuclear factor-6 (*HNF-6*), the prototype onecut family, controls B lymphopoiesis in the fetal liver [53]. The identification of this protein implies that adaptive immunity in zebrafish is activated in the invasion stage. Thrombospondin-1 (*thbs1*) plays an important role in rheumatoid arthritis and it is also reported to be a pro-inflammatory molecule [53]. Its identification in the invasion stage suggests that the host is infected by the pathogen and needs to activate inflammatory responses to recruit immune cells for defense.

**Figure 16:**
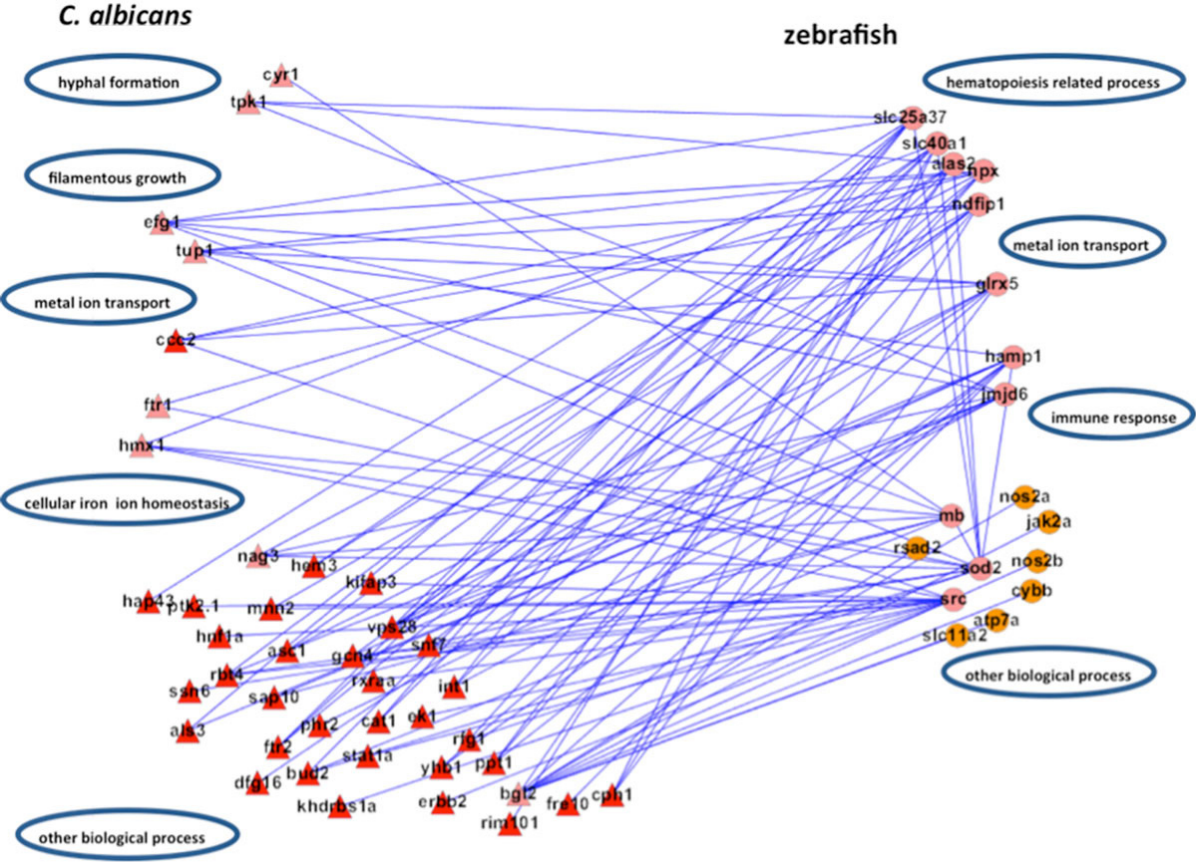
**The sub-network for the iron host-pathogen interaction network in the invasion stage**. There were 271 interspecies interactions between *C. albicans *and zebrafish in the iron host-pathogen interspecies interaction network in the invasion stage. There were nine iron-related virulence proteins identified as significant in *C. albicans *(pink triangles) and 11 iron-related immune proteins in zebrafish (pink circles). The main functional modules for *C. albicans *in the invasion stage were identified to be hyphal formation (*cyr1, tpk1*), metal ion transport (*ccc2*), filamentous growth (*efg1, tup1*), and cellular iron ion homeostasis (*ftr1, hmx1*), while the main functional modules for zebrafish were identified to be the immune response (*hamp1, jmjd6*), metal ion transport (*glrx5*), and hematopoiesis-related processes (*slc25a37, slc40a1, alas2, hpx, ndfip1*).

In contrast to the adhesion stage, during the invasion stage the main aim of *C. albicans *is to form hyphae and to penetrate the host (Figure 16). However, the competition for resources continues, and *C. albicans *appears to have the advantage in the competition in this middle stage due to the damage done to the host epithelial surface during the adhesion stage. At this stage, the host also enhances associated hematopoietic biological processes to keep iron and glucose resources balanced and stable. In further contrast to the previous adhesion stage, filamentous growth and hyphal formation now begin to flourish. The biological processes of metal ion transport for both host and pathogen continue, allowing both to compete for the associated resources. Notably, the immune response of zebrafish is strongly related to the glucose-related proteins. This may suggest that glucose is an important source of energy for the host in activating immune response mechanisms.

### Damage stage

We identified 204 and 286 interspecies interactions between *C. albicans *and zebrafish in iron and glucose competition, respectively.

For the iron competition interspecies network in the damage stage, its robustness was estimated to be 0.8301, which was far larger than the robustness estimated in the glucose competition interspecies network (-0.088). We observed that robustness in the glucose competition interspecies network changes from 1.8281 to -0.0880 in the invasion and damage stage, respectively. This indicates that the glucose dynamic system is unstable and in fact out of control during the damage stage. This may result from the fact that zebrafish PPI networks are severely damaged and normal glucose metabolism is not possible. Interestingly, positive robustness of the iron competition interspecies network indicates that iron competition is still active and that the zebrafish iron competition network can resist intrinsic perturbation with a ratio of 0.8301.

We further identified proteins of statistically significant interactivity in *C. albicans *for the damage stage, comprising nine iron-related virulence proteins (GO annotation: pathogenesis and virulence - 33.3%, hyphal formation - 22.2%, filamentous growth - 11%, metal ion transport - 22.2%) and four glucose-related virulence proteins (GO annotation: hyphal growth - 25%, virulence - 50%) (Table 10, 11, Figure 10, 11). According to the GO annotations, most of these proteins are involved in biological processes related to pathogenesis and filamentous growth. This observation indicates that *C. albicans *begins to increase the destruction of the attachment location and of the entire host organism.

In zebrafish, we identified six iron-related immune proteins (GO annotation: hematopoiesis-related process - 66.7%, immune response - 33.3%) and two glucose-related immune proteins (GO annotation: glucose transport - 50%) (Table 12-13 Figure 12-13). In comparison to the proteins identified in the adhesion and invasion stages, we found many proteins common to the three infection stages, in particular, *alas1, hamp1, jmjd6*, and *tfr1a*, which have been widely shown to be involved in hemopoietic activities. This observation suggests that the host continues sanguification in order to compensate for the loss of iron during the infection.

### Differentially regulated proteins with partial evidence

*Tup1 *was again identified during the damage stage in our host-pathogen interspecies interaction network. This may imply that *tup1 *plays a key role throughout the infection. In the damage stage, *tup1 *should be responsible for hyphae formation. It was also shown that *tup1 *mutation results in pseudo-hyphal morphology [54]. The other identified protein, *phr2*, is reported to be essential for systemic infection of mice in low pH environments only [55]. Specifically, *phr2 *was undetectable at high pH levels in the blood of mice, while it can be detected at pH < 5.5. Its identification in the damage stage suggests that in order to support its own survival and pathogenesis, *C. albicans *needs to absorb more iron from the host, in addition to utilizing the reductase pathway to acquire more iron. This creates an environment that is acidic, leading to the activation of *phr2*. *Hap43 *is reported to be involved in *C. albicans *virulence. The deletion of *hap43 *attenuates the virulence of *C. albicans *in a mouse model [23]. Its identification suggests that the virulence of *C. albicans *is enhanced during the damage stage. The fact that *ftr2 *is active in the damage stage may be due to the higher level of iron availability [45]. In this stage, the iron competition between host and pathogen ceases. *C. albicans *appears to be victorious in the competition for this essential resource, resulting in greater availability of iron for *C. albicans *than during the previous two stages.

In zebrafish, only *edn1 *was identified in the glucose interspecies interaction network in the damage stage. Endothelin-1 (*edn1*) is reported to be capable of stimulating glucose transport in the target tissues of insulin [56]. Its presence suggests that although the zebrafish may eventually succumb to the infection, glucose as a fundamental element for maintaining life is still needed by the host.

**Figure 17:**
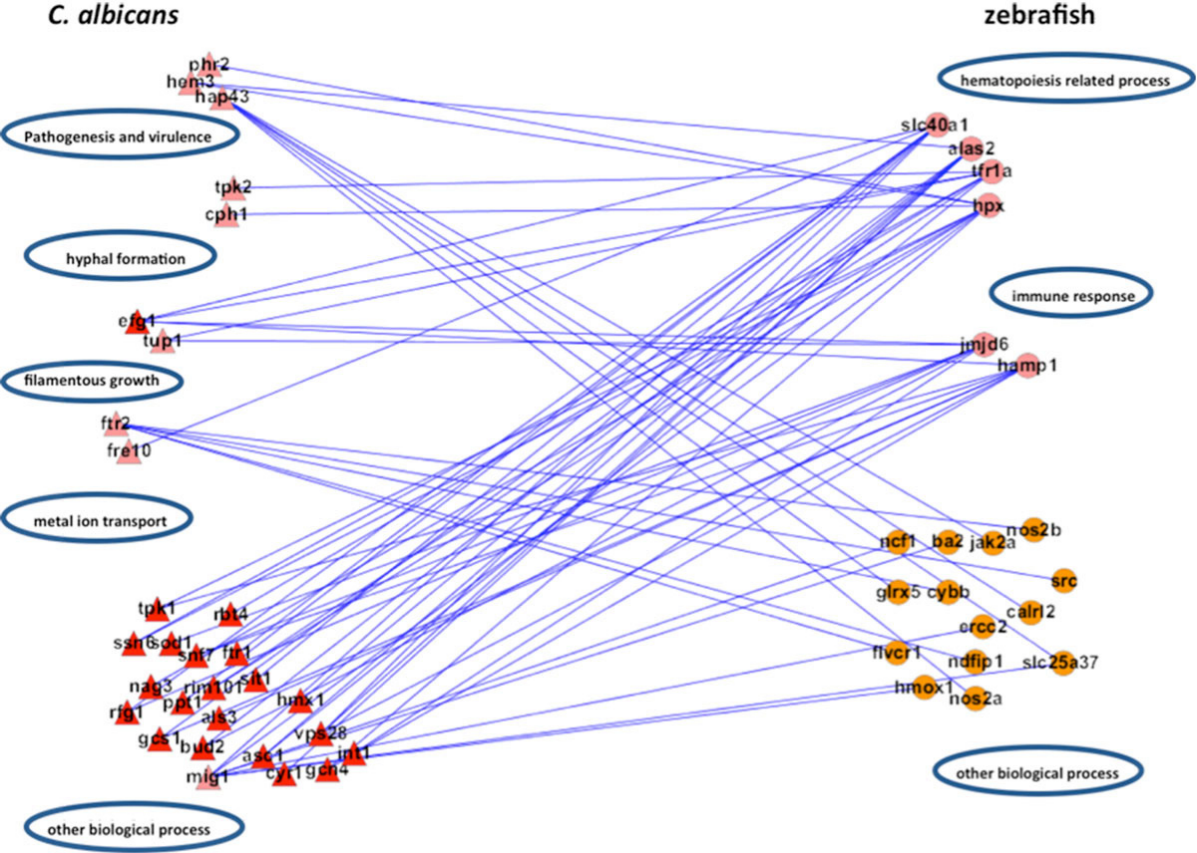
**The sub-network for the iron host-pathogen interspecies interaction network in the damage stage**. There were 294 interspecies interactions between *C. albicans *and zebrafish in the iron host-pathogen interspecies interaction network in the damage stage. There were nine iron-related virulence proteins identified as significantly interactive in *C. albicans *(pink triangles) and six iron-related immune proteins in zebrafish (pink circles). The main functional modules for *C. albicans *in the damage stage were identified to be pathogenesis and virulence (*ph32, hem3, hap43*), metal ion transport (*ftr2, fre10*), hyphal formation (*tpk2, cph1*), and filamentous growth (*efg1, tup1*), while the main functional modules for zebrafish were identified to be the immune response (*hamp1, jmjd6*) and hematopoiesis-related processes (*slc40a1, alas2, tfr1a, hpx*).

During the damage stage (Figure 17), the pathogen strengthens its pathogenic attack by inducing filamentous growth. The transportation of iron continues. Although the host appears to have no other strategies to defend against the invader, it however still maintains the production of essential resources to prolong its own survival.

From the engagement in the previous two stages, *C. albicans *successfully infects the zebrafish and continues to damage the host. However, while the zebrafish's system is now much weaker, it still seeks to remain viable by continuing the hematopoiesis process (a fundamental process for any biological organism).

The above observations and predictions derived from the constructed host-pathogen interspecies interaction networks allowed us to explore the dynamics and underlying mechanisms by which *C. albicans *interacts with zebrafish during infection, providing new insights for future experiments in infectious diseases.

## Discussion

During infection, the competition for iron and glucose plays a critical role in the battle between host and pathogen. Our estimation of robustness of the competition interspecies networks between *C. albicans *and zebrafish assists in elucidating the offensive and defensive mechanisms adopted by both organisms.

### *C. albicans*, as an aggressor

The strategies of *C. albicans *for the competition for iron have evolved to high efficiency. These strategies can be related to three crucial iron acquisition sub-systems: (i) siderophore uptake, (ii) hemoglobin degradation, and (iii) a reductive pathway system. We can also use the estimation of the robustness to observe the relationship between three infection stages from a systematic point-of-view to discover the focus of resource competition as an offensive strategy for *C. albicans*.

In the early adhesion stage, robustness for the iron interspecies competition network is 0.7274. Compared with the robustness of the unstable glucose interspecies competition network (-0.5306), iron interspecies competition network is more robust to withstand the intrinsic perturbation such as thermal fluctuation or other process noise. This indicates that *C. albicans *conducts systematic iron collection during the early adhesion stage. During the adhesion stage, the primary goal of *C. albicans *is to adhere to the epithelial cell surface of the host tissue. Among the proteins that were identified in our iron host-pathogen interspecies interaction networks, *tup1 *and *asc1 *were related to attachment [57,58]. *C. albicans *seems to begin to detect the iron in its surroundings at this stage. These iron resources, however, are often in the form of ferric iron (Fe^3+^) in the host organism rather than the soluble ferrous form (Fe^2+^). *C. albicans *cannot use ferric iron directly, and its activation of the reductive pathway system is necessary to reduce ferric iron to the ferrous form. This activation can be inferred from the network during the adhesion stage, where the protein *fre10 *(previously reported to be highly related to the reductive activity [21]) was identified. The reductive pathway system is also involved in multicopper oxidase activity, which prevents the production of toxic free radicals from the reductive process during the transition of electrons by oxidizing ferrous iron to ferric iron. We also identified the copper transporter *ccc2*, which is known to be involved in multicopper oxidase activity [59]. Aside from acquiring hemoglobin from its surroundings, it is also necessary for *C. albicans *to activate the relevant receptors. The well-known ferritin receptor, *als3*, was also identified but was found to be much more active in the damage stage than the adhesion stage. This may suggest that the acquisition of ferritin occurs mainly during the latter stage since ferritin is more stable than other types of iron proteins [60]. Further investigation of the numbers of linkages in the adhesion stage (see Additional file 1.xlsx in supplementary file for details) revealed that the hemoglobin-receptor gene family *rbt4 *had the maximum number of linkages. This may suggest that during early infection, the initial strategy of *C. albicans *is to activate all receptors involved in the detection of ferritin, transferrin, and hemoglobin, to detect possible sources of iron in its surroundings. If ferric iron is found to be the major iron resource available, the reductive pathway system is then activated to convert ferric iron to the usable ferrous form. It can be inferred from the network that *C. albicans *is mainly involved in adherence and detection of the iron sources in its surroundings during the adhesion stage, preparing the pathogen for the following invasion and damage stage. Similar to iron detection, *C. albicans *also begins sensing for glucose for possible uptake. *Hgt4 *and *gpa2 *were identified in our glucose network as involved in glucose detection [21,22]. Furthermore, *snf1 *was identified as involved in cell adhesion [61]. The identification of this glucose-related protein suggests that glucose is also an essential resource for adhesion activity in *C. albicans*.

In the next stage of the infection process, normally known as the invasion stage, the robustness value of the iron competition interspecies network is negative (-0.4143) while it is positive in the glucose competition interspecies network (1.8281). This demonstrates that the competition for glucose might be more important than iron during the invasion stage. When invasion starts, *C. albicans *simultaneously begins hyphal formation and filamentous growth, penetrating the host tissue. From our constructed glucose interspecies network, the protein *hgt4, tps1, gpa2*, and *rim20 *were identified to be involved in hyphal formation and virulence. Since hyphal transition is the main focus for *C. albicans *at this stage, these glucose-related proteins are needed to support its development. The positive value of the robustness for the glucose competition network (1.8281) indicates that *C. albicans *needs stable growth of mycelium by preying glucose from zebrafish. And it infers the glucose is the main target and more relevant during the invasion stage. On the other hand, an adequate supply of iron is also necessary for its invasion and survival. Iron obtained via the reductive pathway is often bound to the high-affinity iron permease and then transported into the cell of the pathogen for further utilization or storage. The protein *ftr1 *(which is reported to play an important role in this transportation and storage [56]) was identified in the iron interspecies interaction network. Efficient use and transportation of iron resources obtained from the adhesion stage are therefore crucial.

In the last (damage) stage of the infection process, iron acquisition by *C. albicans *is reduced since the iron acquired from the host almost reaches saturation for the pathogen at this point. The positive robustness value (0.8301) of the iron competition interspecies network indicates that *C. albicans *now stably focuses on the storage of the iron obtained during the first two stages. Compared with iron competition interspecies network, robustness of the glucose competition interspecies network is negative (-0.0880), indicating a unstable system. This suggests that *C. albicans *focus on iron competition instead of glucose competition in the last damage stage, where zebrafish cells are severely damaged and depriving irons from the damaged zebrafish cells become the primary goal for *C. albicans*.

As the aggressor in the iron and glucose competition, *C. albicans *was found to execute well-planned strategies, as can be inferred from the significantly interactive proteins identified in the networks. In the early stage of infection, the pathogen mainly focuses on adhering to the epithelial surface of the host and detecting the available iron sources in its surroundings. In the invasion stage, *C. albicans *begins harvesting glucose to support its filamentous growth and hyphal formation, as well as transporting and storing associated resources from the zebrafish. In the final damage stage, *C. albicans *enhances its attack to finally overcome the host in this competition for resources.

### Zebrafish, as a defender

In the zebrafish host, we identified several proteins (*alas2, tfr1a, slc25a37*) that are associated with heme synthesis and hematopoiesis in the three constructed host-pathogen interspecies interaction networks throughout the infection. This may imply that heme synthesis and the hematopoiesis process are upregulated during the infection process to inhibit iron deprivation by *C. albicans*. As reductase activity is involved in both pathogen and host during iron competition, we also identified *glrx5 *(reported to be responsible for the mobilization of electrons [30]) in both the adhesion and invasion stage. When the pathogen adheres to the surface of the host cell, the relevant pattern recognition receptors in zebrafish are induced to produce the precise antimicrobial peptides required to defend against the pathogen. In our adhesion stage networks, *hamp1 *was identified as being involved in this process [62].

In the invasion and damage stage, the zebrafish attempts to produce more iron to counteract the iron deprivation induced by *C. albicans*. Accordingly, several proteins associated with hematopoiesis (*alas2, hamp1, slc40a1, slc25a37, tfr1a*, and *hpx*) were also identified. Interestingly, robustness analysis for the two stages reveal that iron competition interspecies networks are unstable during the invasion stage but stable during the damage stage. This could infer that the activities associated with hematopoiesis are stronger during the damage stage since zebrafish is now severely damaged.

In investigating significantly interactive proteins in zebrafish during the three distinct infection stages, we observed that the zebrafish maintained almost the same biological process of hematopoiesis in order to prolong its life as much as possible.

As a defender in this competition for resources, zebrafish are passive and responsive. Even though the host is losing essential resources, it has no defensive strategies beyond enhancing the production of these resources to keep its own biological processes balanced and stable.

With respect to glucose competition, several identified proteins were associated with immunity (*gpia, hif1ab*, and *ins *in the adhesion stage; *dbh, gpia, hif1ab, onecutl*, and *thbs1 *in the invasion stage). The identification of these glucose-related immune proteins indicates that in addition to engaging in competition for iron and glucose, zebrafish also activate their immune system to combat the intruder. We observe that the not only the robustness of glucose competition interspecies network in the invasion stage reaches its peak, but also most of immune-related proteins are identified in the invasion stage than in the other two stages. This strongly suggests that the major defense of the host primarily occurred during the middle stage of the infection, when *C. albicans *initiated hyphal formation and filamentous growth.

## Conclusion

Our proposed model provides a novel way to explore and predict how these two organisms interact during infection, employing the construction of host-pathogen interspecies interaction networks and use of simultaneously quantified time-course microarray data. Moreover, the robustness analysis provided a quantitative measure of the intrinsic perturbation tolerance ability of a competition network for each infection stage. This helps elucidate the resource competition mechanisms underlying the infection between *C. albicans *and zebrafish. We identified several proteins with statistically significant interactions during the three infection stages (adhesion stage: nine iron-related and four glucose-related virulence proteins in *C. albicans*, 10 iron-related and three glucose-related immune proteins in zebrafish; invasion stage: nine iron-related and seven glucose-related virulence proteins, 11 iron-related and seven glucose-related immune proteins; damage stage: nine iron-related and four glucose-related virulence proteins, six iron-related and two glucose-related immune proteins). Combining these useful tools, we provided insights to elucidate the offensive and defensive mechanisms of *C. albicans *and zebrafish during their competition for iron and glucose.

## Competing Interests

The authors declare that they have no competing interests.

## Authors' contributions

Conceived and designed the experiments: YJC CYL. Performed the experiments: YJC CYL. Analyzed the data: CNL CL YCW FYL WPH BSC. Contributed reagents/materials/analysis tools: CL CNL YCW FYL YWC BSC. Wrote the manuscript: CL CNL

## Supplementary Material

Additional file 1 **GO annotations of top 10 hub proteins ranked by degree in glucose and iron PPIN for adhesion, invasion and damage stage**. Click here for file

Additional file 2 **Provides the figure files used in the draft**. Click here for file

Additional file 3 **The glucose and iron protein-protein interaction network (PPIN) for the adhesion, invasion and damage stage**. Click here for file

Additional file 4 **Differentially regulated proteins with strong evidence in glucose and iron PPIN for adhesion, invasion and damage stage**. Click here for file

## Declarations

The publication of this supplement was funded by the National Science Council of Taiwan, R.O.C. with funding number: NSC-100-2627-B-007-004.

This article has been published as part of *BMC systems Biology *Volume 8 Supplement 5, 2014: Proceedings of the 25th International Conference on Genome Informatics (GIW/ISCB-Asia): Systems Biology. The full contents of the supplement are available online at http://www.biomedcentral.com/bmcsystbiol/supplements/8/S5.
