# Supplementary figures and images for: Robustness analysis on interspecies interaction network for iron and glucose competition between Candida albicans and zebrafish during infection
Source: BMC Syst Biol. 2014 Dec 12;8(Suppl 5):S6. doi: 10.1186/1752-0509-8-S5-S6 (PMC4305985; doi:10.1186/1752-0509-8-S5-S6)

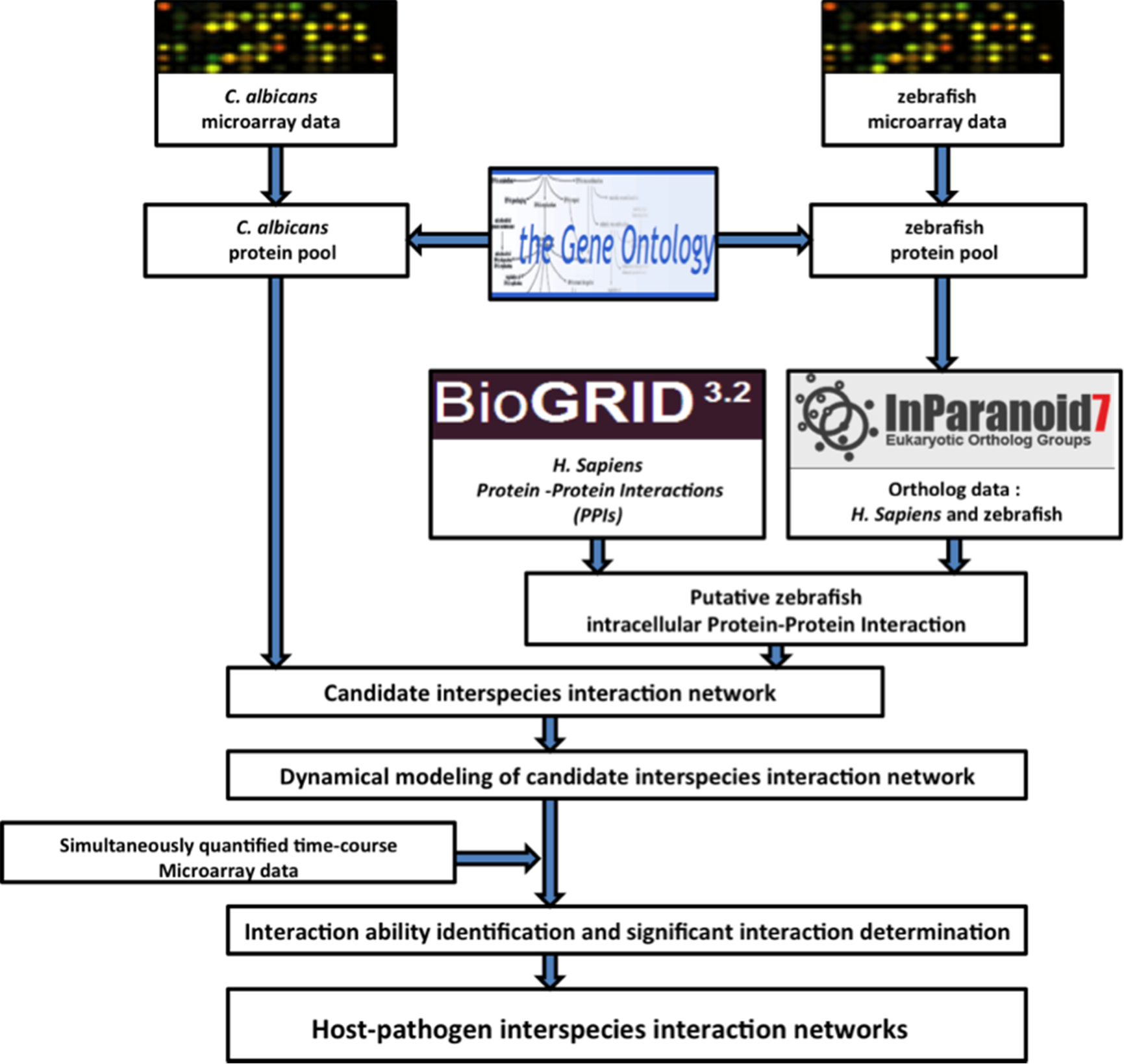

Supplement: Additional file 2 — Provides the figure files used in the draft. [file 1752-0509-8-S5-S6-S2.zip › Additional file 1/Figure 1.png]

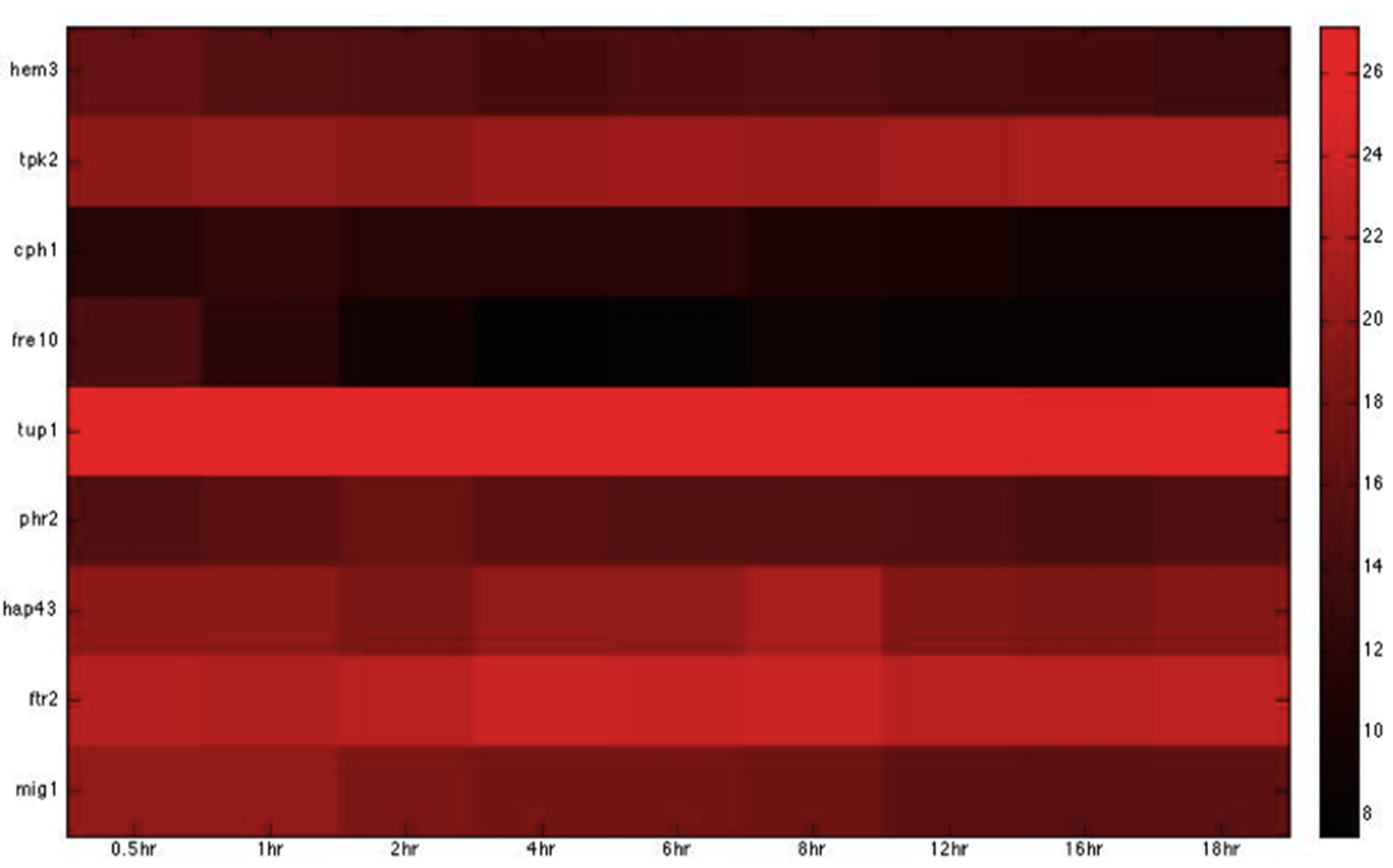

Supplement: Additional file 2 — Provides the figure files used in the draft. [file 1752-0509-8-S5-S6-S2.zip › Additional file 1/Figure 10.png]

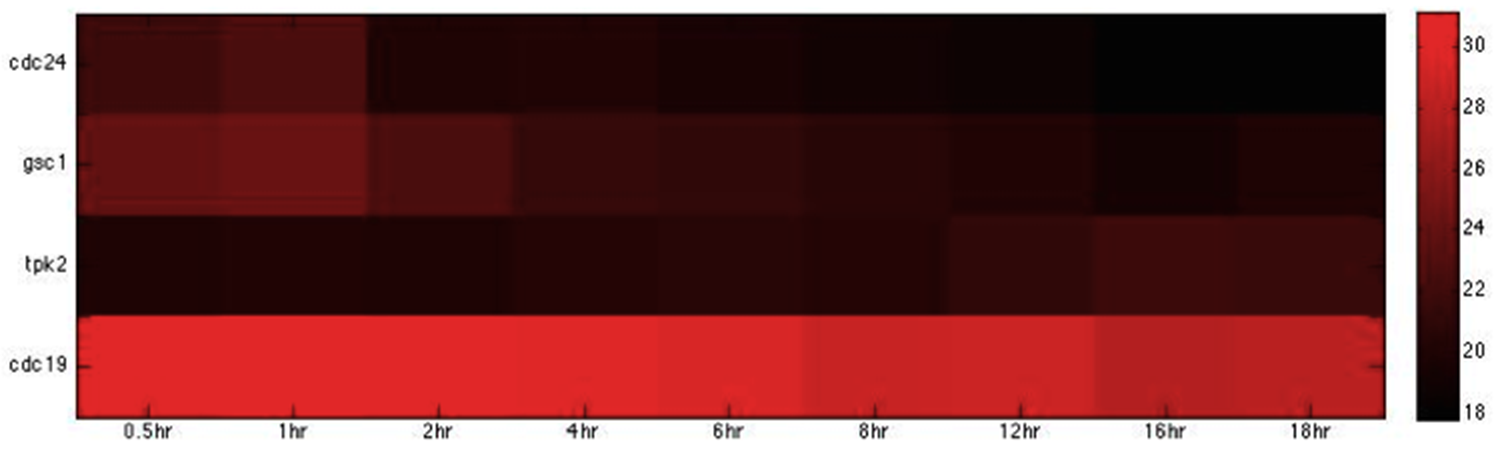

Supplement: Additional file 2 — Provides the figure files used in the draft. [file 1752-0509-8-S5-S6-S2.zip › Additional file 1/Figure 11.png]

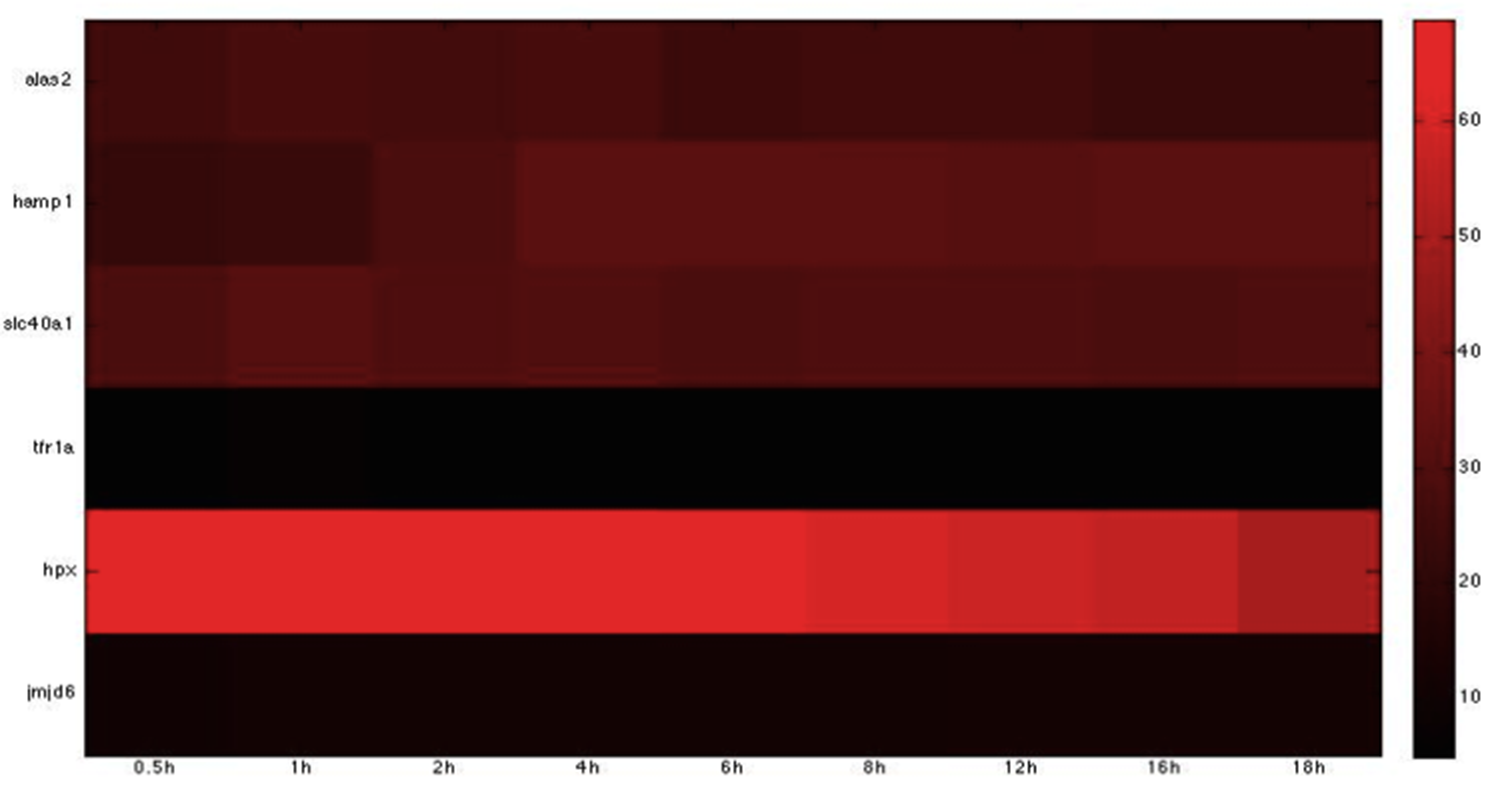

Supplement: Additional file 2 — Provides the figure files used in the draft. [file 1752-0509-8-S5-S6-S2.zip › Additional file 1/Figure 12.png]

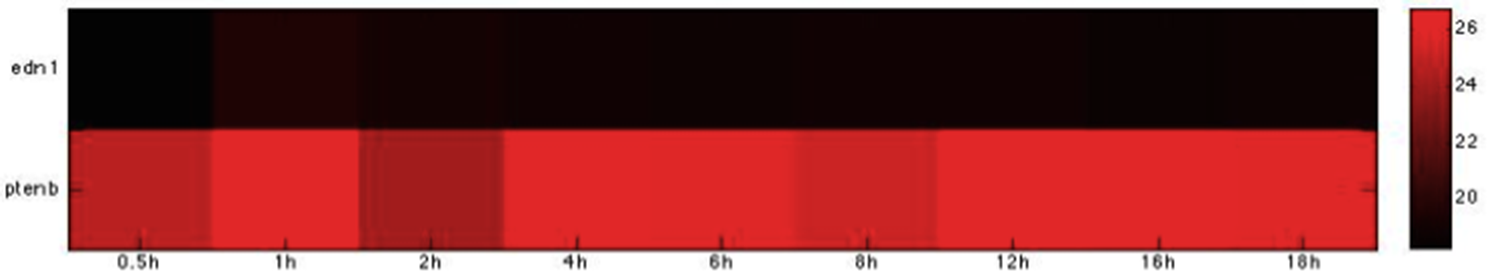

Supplement: Additional file 2 — Provides the figure files used in the draft. [file 1752-0509-8-S5-S6-S2.zip › Additional file 1/Figure 13.png]

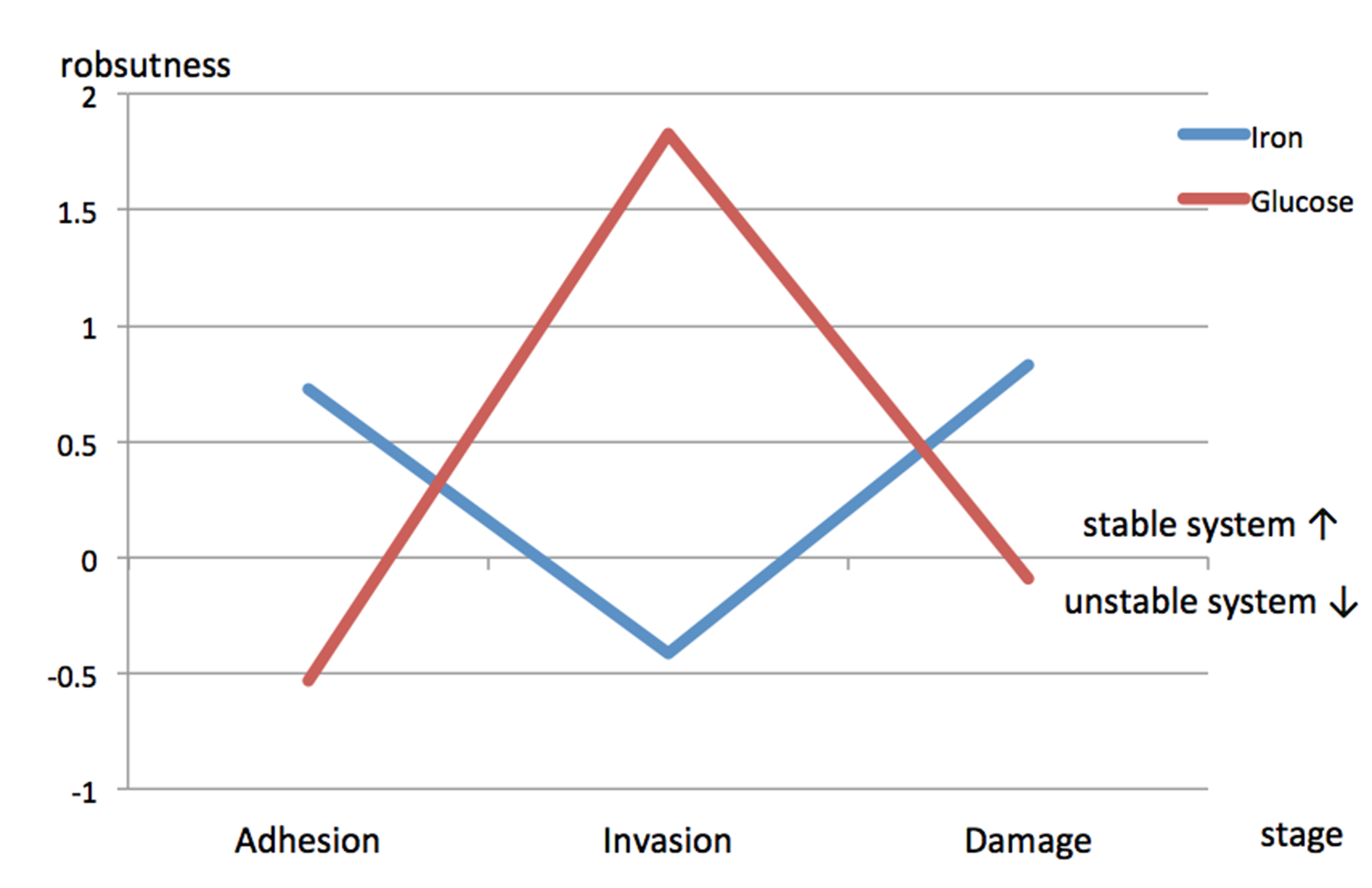

Supplement: Additional file 2 — Provides the figure files used in the draft. [file 1752-0509-8-S5-S6-S2.zip › Additional file 1/Figure 14.png]

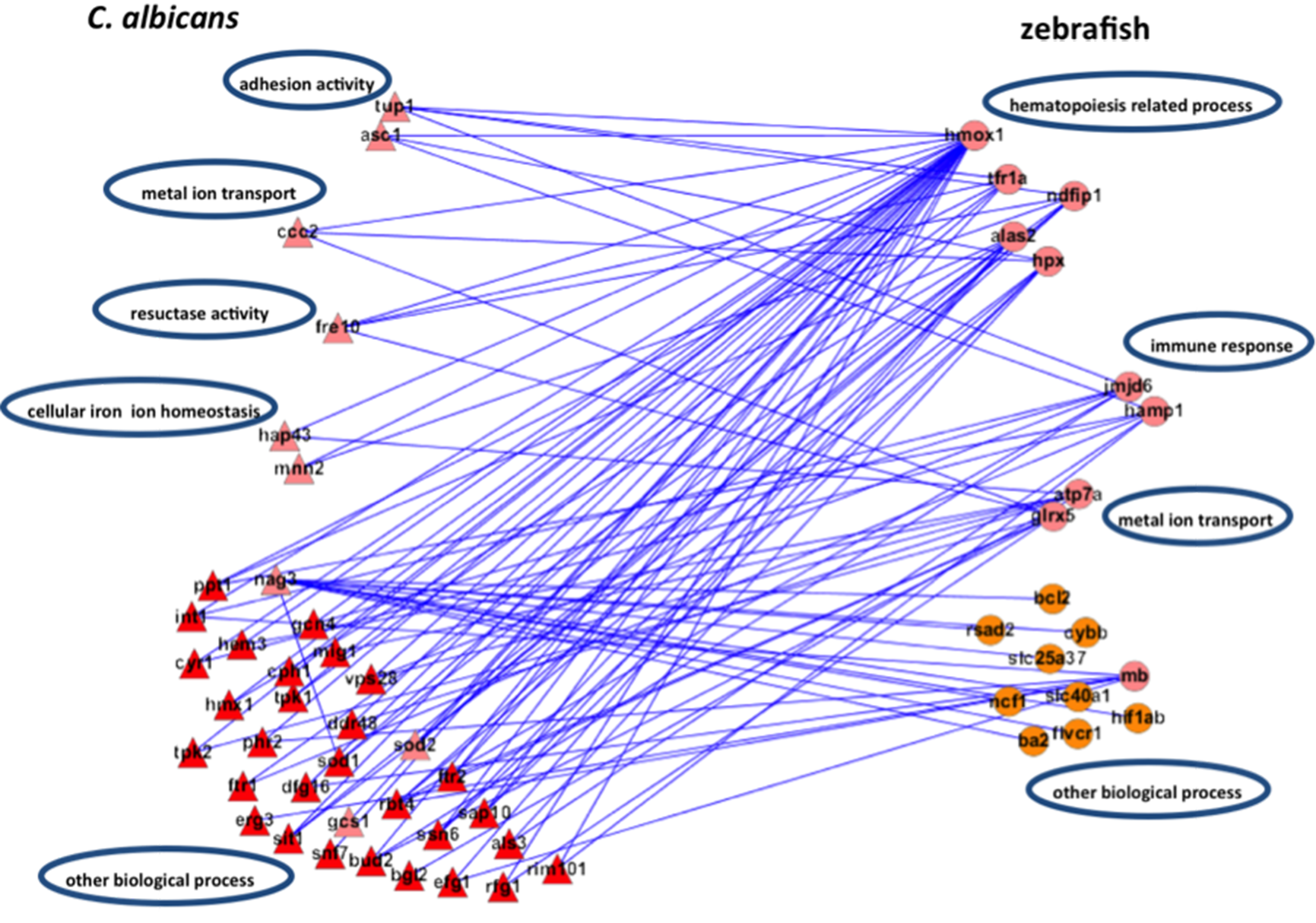

Supplement: Additional file 2 — Provides the figure files used in the draft. [file 1752-0509-8-S5-S6-S2.zip › Additional file 1/Figure 15.png]

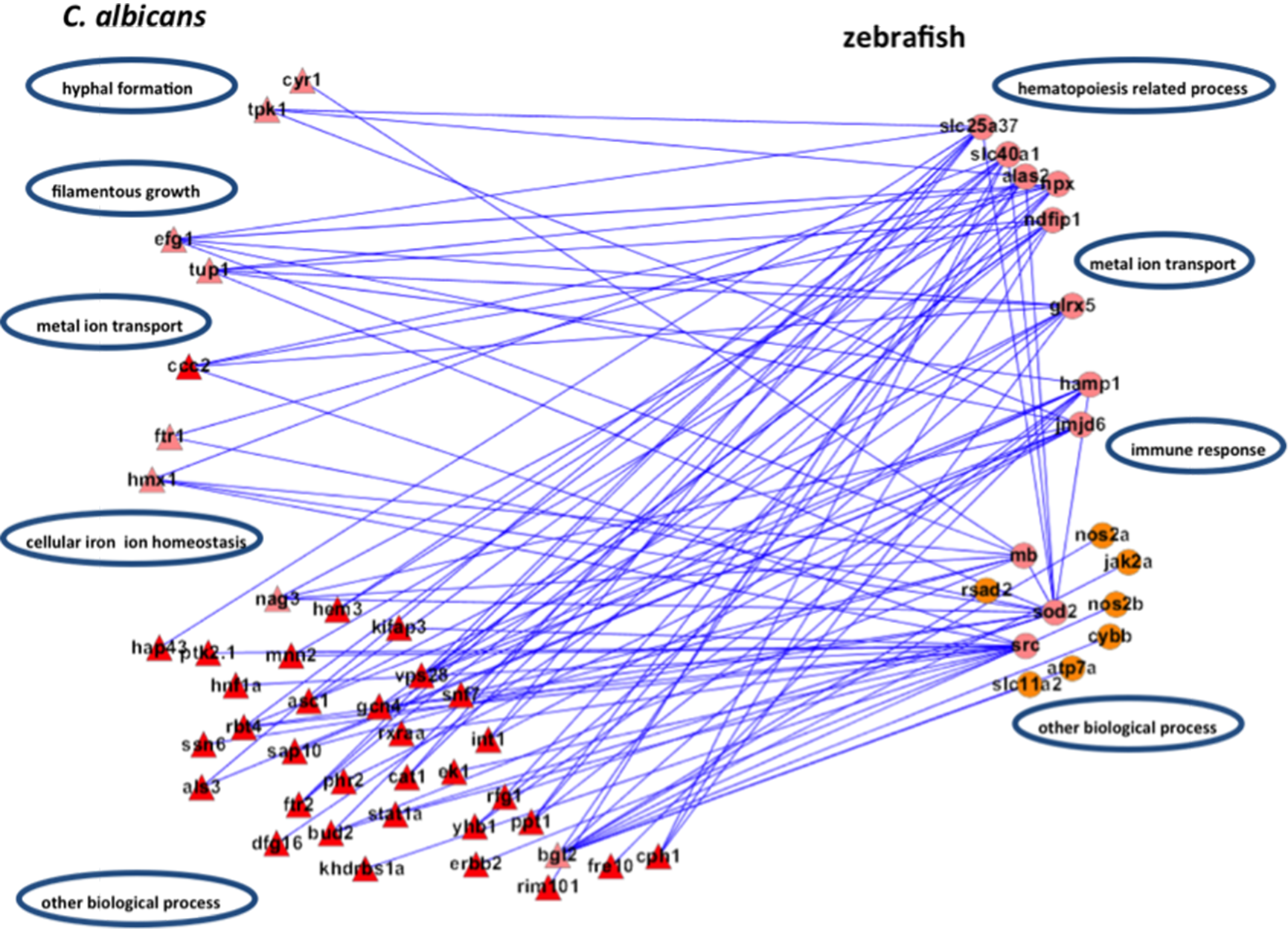

Supplement: Additional file 2 — Provides the figure files used in the draft. [file 1752-0509-8-S5-S6-S2.zip › Additional file 1/Figure 16.png]

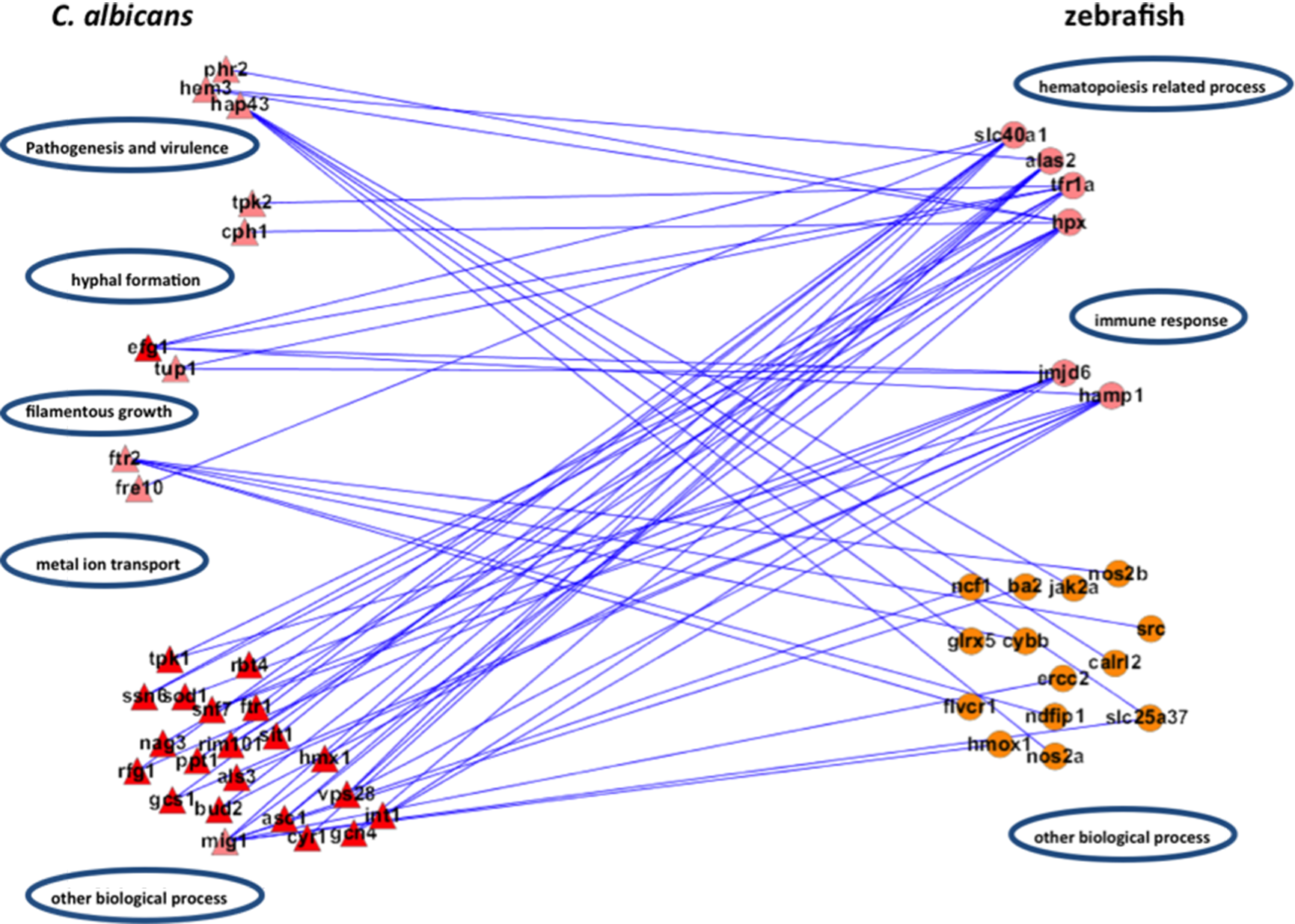

Supplement: Additional file 2 — Provides the figure files used in the draft. [file 1752-0509-8-S5-S6-S2.zip › Additional file 1/Figure 17.png]

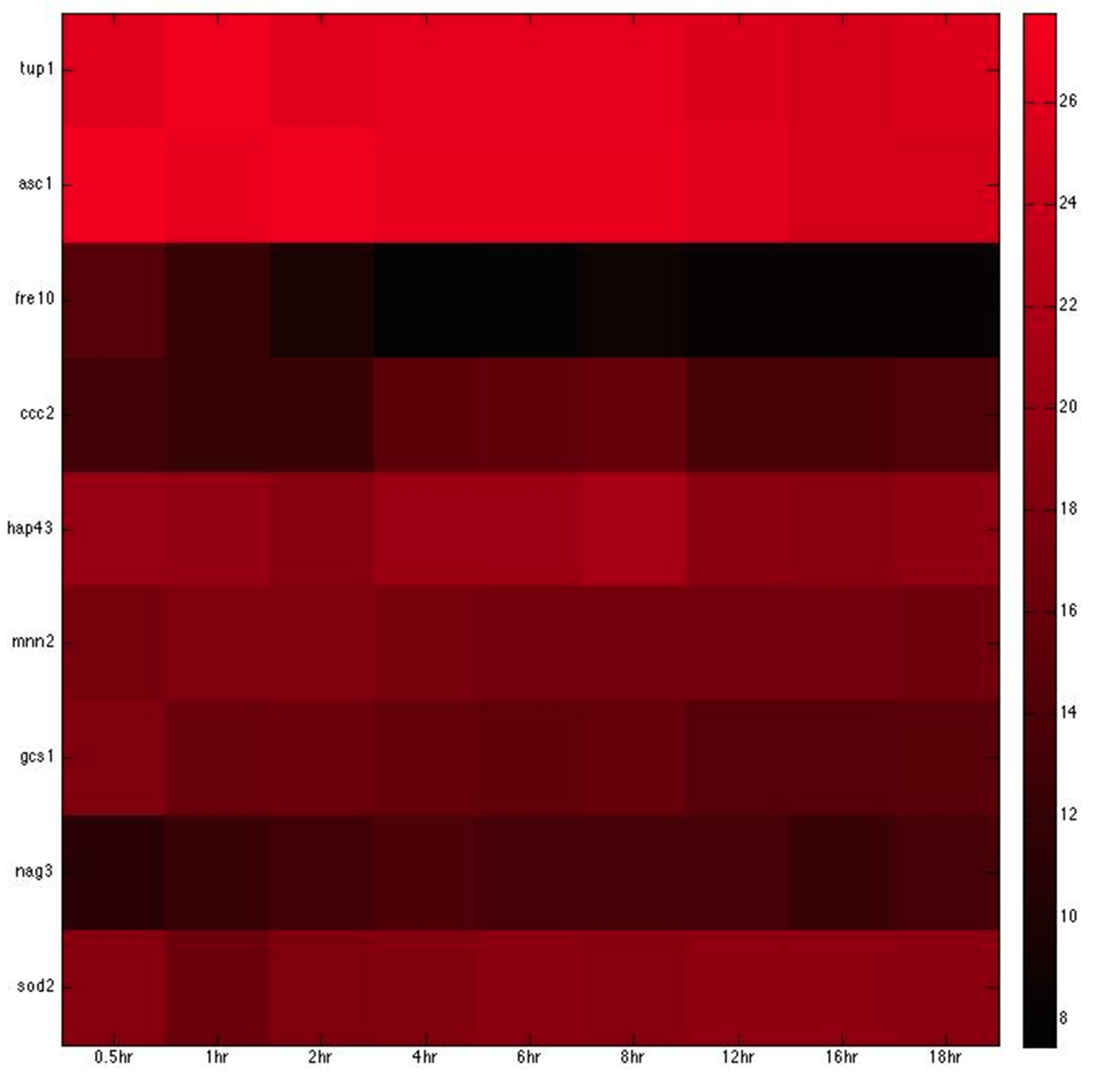

Supplement: Additional file 2 — Provides the figure files used in the draft. [file 1752-0509-8-S5-S6-S2.zip › Additional file 1/Figure 2.png]

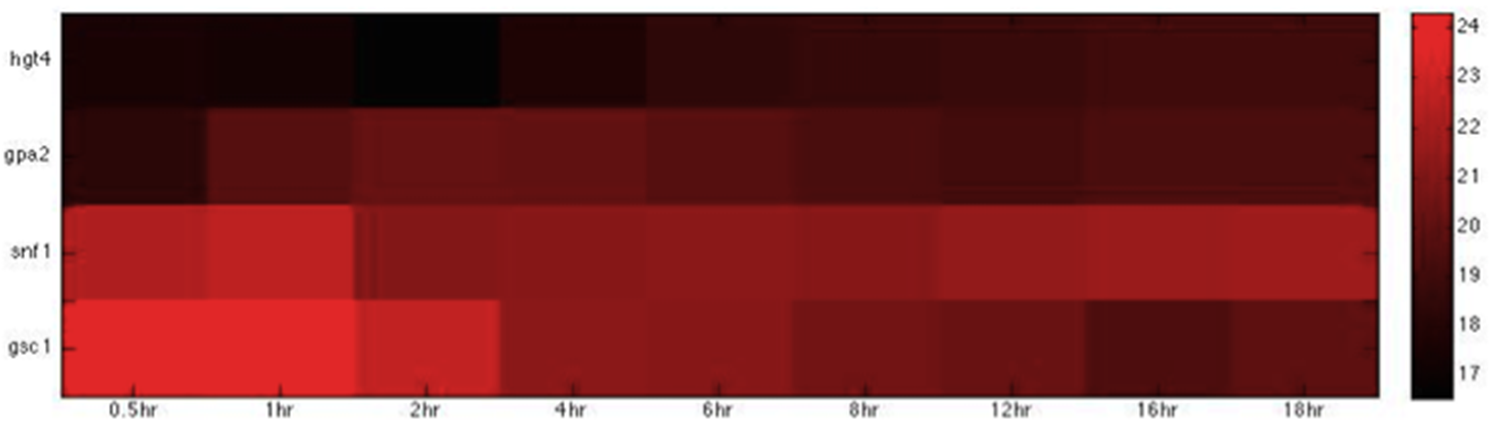

Supplement: Additional file 2 — Provides the figure files used in the draft. [file 1752-0509-8-S5-S6-S2.zip › Additional file 1/Figure 3.png]

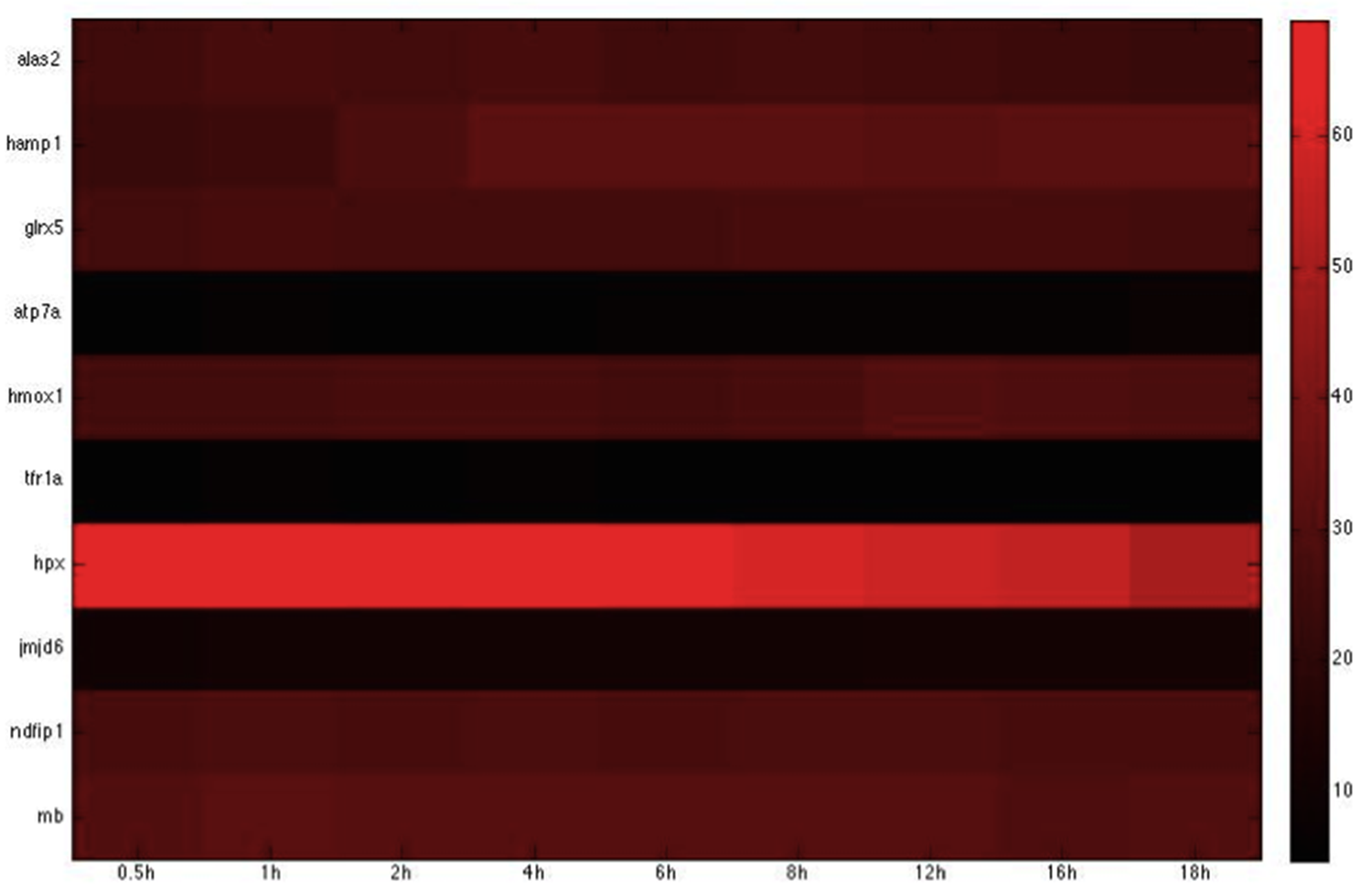

Supplement: Additional file 2 — Provides the figure files used in the draft. [file 1752-0509-8-S5-S6-S2.zip › Additional file 1/Figure 4.png]

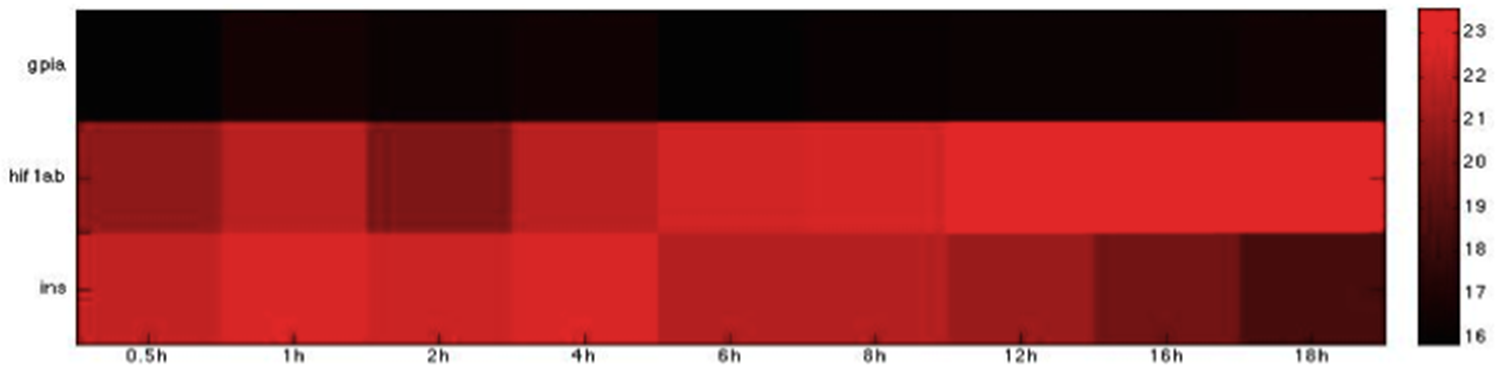

Supplement: Additional file 2 — Provides the figure files used in the draft. [file 1752-0509-8-S5-S6-S2.zip › Additional file 1/Figure 5.png]

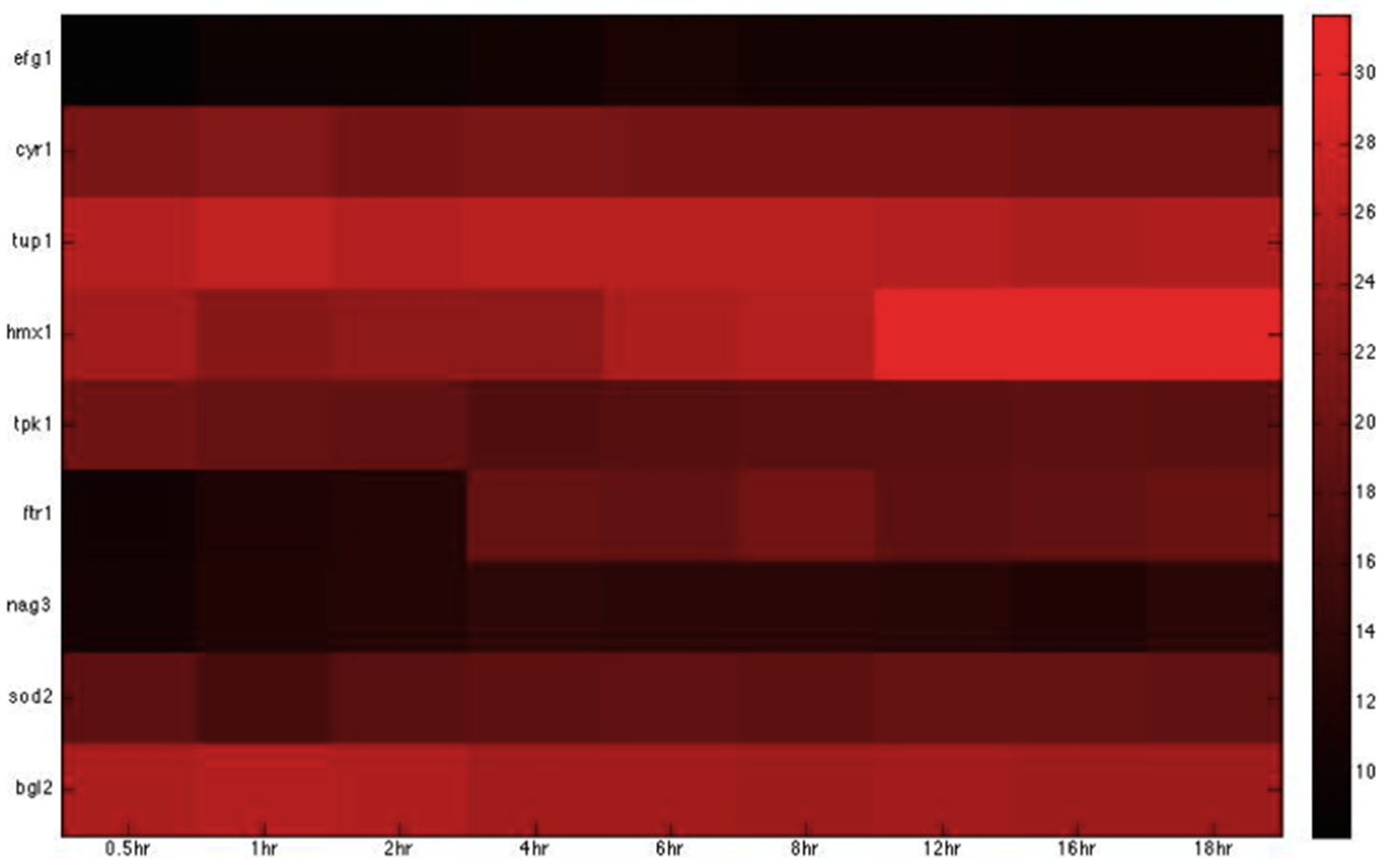

Supplement: Additional file 2 — Provides the figure files used in the draft. [file 1752-0509-8-S5-S6-S2.zip › Additional file 1/Figure 6.png]

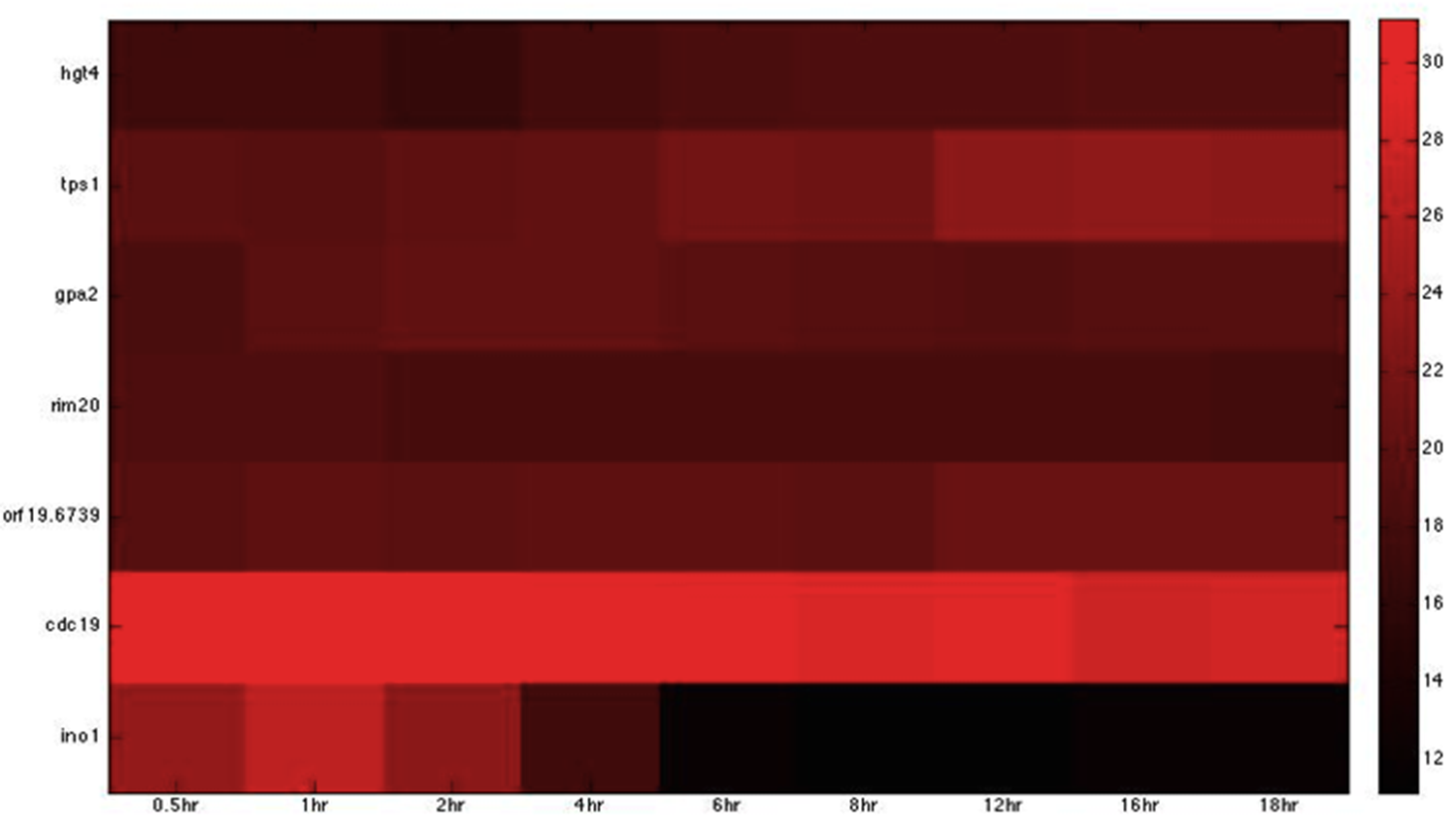

Supplement: Additional file 2 — Provides the figure files used in the draft. [file 1752-0509-8-S5-S6-S2.zip › Additional file 1/Figure 7.png]

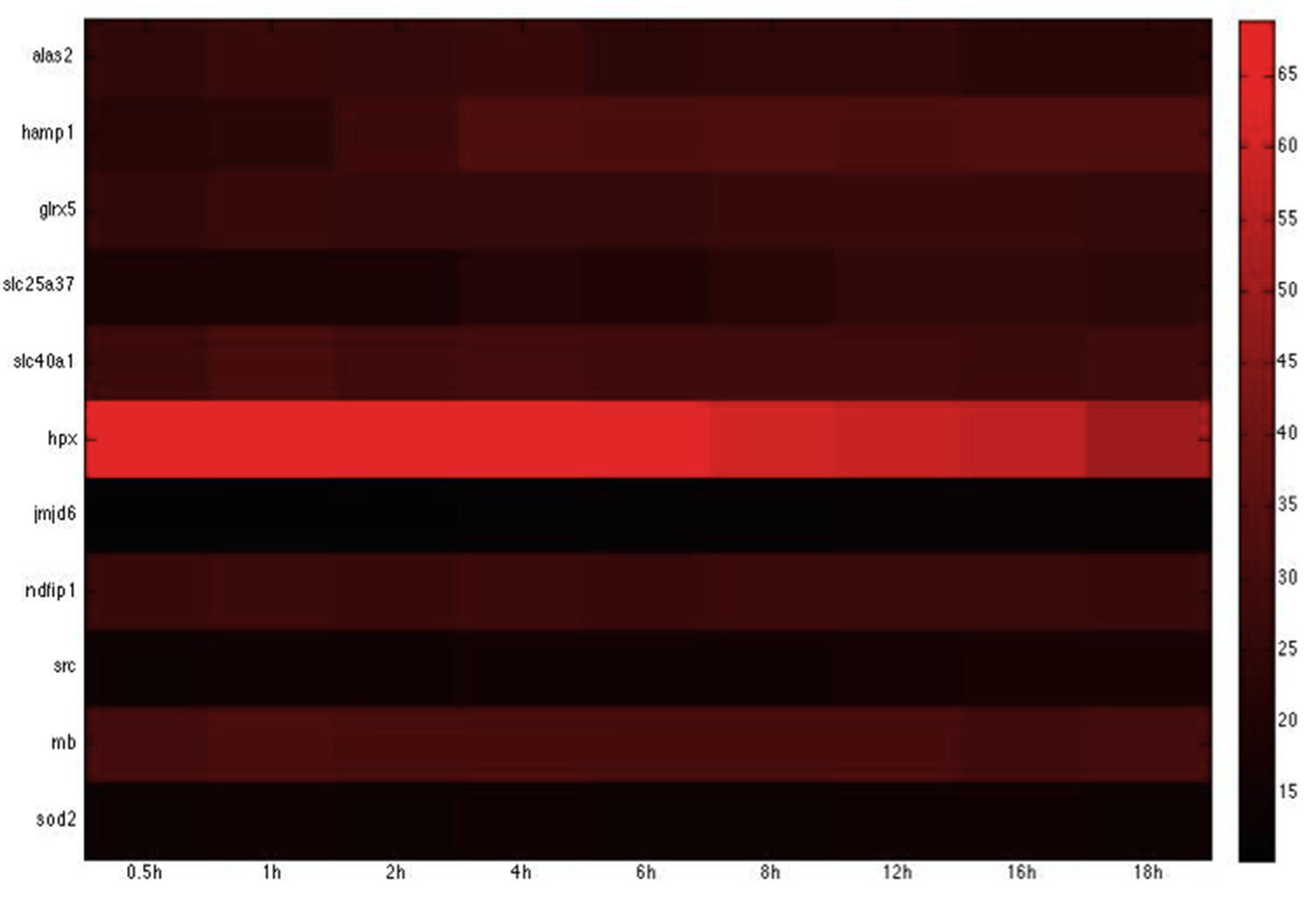

Supplement: Additional file 2 — Provides the figure files used in the draft. [file 1752-0509-8-S5-S6-S2.zip › Additional file 1/Figure 8.png]

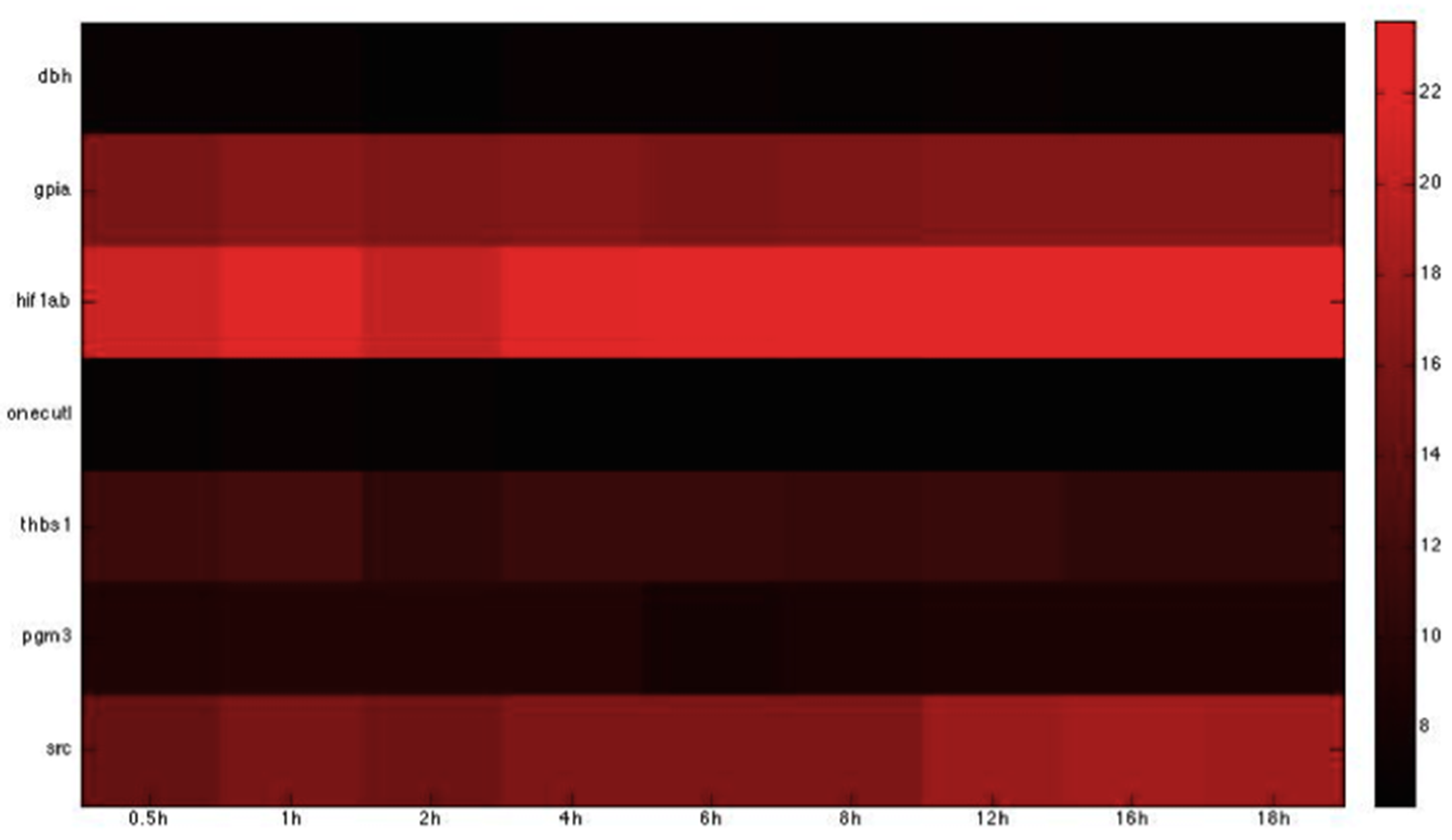

Supplement: Additional file 2 — Provides the figure files used in the draft. [file 1752-0509-8-S5-S6-S2.zip › Additional file 1/Figure 9.png]
