# Supplementary material for: Robustness analysis on interspecies interaction network for iron and glucose competition between Candida albicans and zebrafish during infection
Source: BMC Syst Biol. 2014 Dec 12;8(Suppl 5):S6. doi: 10.1186/1752-0509-8-S5-S6 (PMC4305985; doi:10.1186/1752-0509-8-S5-S6)
Supplement: Additional file 4 — Differentially regulated proteins with strong evidence in glucose and iron PPIN for adhesion, invasion and damage stage. [file 1752-0509-8-S5-S6-S4.docx]

**Adhesion stage**

**Differentially regulated proteins with strong evidence**

In *C. albicans*, *tup1* and *asc1* were identified in our iron host-pathogen interspecies interaction network during the early adhesion stage. *tup1* is a transcriptional regulator of hyphal transition. Mutation of *tup1* has been shown to result in the formation of only pseudohyphae and thus affect its ability to adhere to and injure epithelial cells [[1](#_ENREF_28)]. The deletion of *asc1* in *C. albicans* resulted in defective adhesion and decreased virulence; furthermore, *C. albicans* with *asc1* mutations was found to be avirulent in a mouse model [[2](#_ENREF_29)]. Thus, the identification of *asc1* in the adhesion stage confirms the validity of our proposed method. Focusing on competition for glucose, the glucose-related proteins *hgt4* and *gpa2* were identified. *hgt4* is related to sugar detection and *hgt4* mutants have been found to cause defective growth even at high sugar concentrations [[3](#_ENREF_30)]. Several findings suggest that *gpa2* is responsible for sensing glucose via a cAMP-dependent mechanism [[4](#_ENREF_31)]. Identifying *hgt4* and *gpa2* indicates that at the adhesion stage, *C. albicans* turns on the detection of glucose. Furthermore, the glucose-related protein *snf1* identified here has been demonstrated to be involved in cell adhesion by a pathway involving the *Snf1-Gal83* form, *FLO11*, and the *Nrg* repressor [[5](#_ENREF_32)]. Its identification in the adhesion stage further validates our proposed method.

For zebrafish, *alas2* and *hamp1* were identified in the adhesion stage in our iron host-pathogen interaction network. *alas2* is involved in the heme biosynthesis process, and it results in congenital sideroblastic anemia in humans [[6](#_ENREF_33)]. *hamp1*, a hepcidin antimicrobial peptide 1, was identified as a pivotal functional protein for defense against bacteria [[7](#_ENREF_34)]. The identification of this antimicrobial peptide at the adhesion stage suggests that the host has indeed detected the presence of a pathogen and has activated the relevant defense mechanisms to protect against the intruder.

| relationship | *C. albicans*  iron-related virulence protein | relationship | *C. albicans*  glucose-related virulence protein |
| --- | --- | --- | --- |
| strong/partial | *tup1* | **strong** | *hgt4* |
| strong | *asc1* | **strong** | *gpa2* |
| partial | *fre10* | **strong** | *snf1* |
| partial | *ccc2* | **unknown** | *gsc1* |
| partial | *hap43* |  |  |
| partial | *mnn2* |  |  |
| unknown | *gcs1* |  |  |
| unknown | *nag3* |  |  |
| unknown | *sod2* |  |  |

| relationship | Zebrafish  iron-related immune protein | relationship | Zebrafish  glucose-related immune protein |
| --- | --- | --- | --- |
| strong | *alas2* | **partial** | *gpia* |
| strong | *hamp1* | **partial** | *hif1ab* |
| partial | *glrx5* | **partial** | *ins* |
| partial | *atp7a* |  |  |
| partial | *hmox1* |  |  |
| partial | *tfr1a* |  |  |
| partial | *hpx* |  |  |
| partial | *jmjd6* |  |  |
| partial | *ndfip1* |  |  |
| unknown | *mb* |  |  |

**Table 1. We list the statistically significant proteins identified in the adhesion stage with C. albicans and zebrafish as well as iron- and glucose-related classification.**

**Invasion stage**

**Differentially regulated proteins with strong evidence**

In *C. albicans*, we identified *efg1* and *cyr1* from the iron host-pathogen interspecies interaction network in the invasion stage. *Efg1* is an important protein for the hyphal formation and filamentous growth of *C. albicans*, as a reduced *efg1* expression level suppresses hyphae formation while over-expression results in enhanced filamentous growth [[8](#_ENREF_53)]. We also identified *cyr1* in the invasion stage, which is not only related to *C. albicans* filamentous growth but also to hyphae formation [[9](#_ENREF_54)]. *Cyr1* is detected in the hyphae form rather than the yeast form [[10](#_ENREF_55)]. The identification of both these proteins in the invasion stage validates our proposed method.

For the glucose interaction network, *hgt4*, *tps1*, and *gpa2* were identified. *hgt4* is known to be involved in the detection of sugar and in glucose transportation [[11](#_ENREF_56)]. This protein affects the morphological transition from yeast to hyphae; *hgt* mutants are less virulent in a mouse model [[3](#_ENREF_30)]. The transition from yeast to hyphae has been reported as impaired for *tps1* mutants [[12](#_ENREF_57)]. *Gpa2* encodes a G-protein alpha-subunit homolog and plays a key role in hyphal formation. The deletion of this protein results in defects in morphological transition [[13](#_ENREF_58)]. The identification of these proteins in the invasion stage again validates our proposed method.

| relationship | *C. albicans*  iron-related virulence protein | relationship | *C. albicans*  glucose-related virulence protein |
| --- | --- | --- | --- |
| strong | *efg1* | **strong** | *hgt4* |
| strong | *cyr1* | **strong** | *tps1* |
| partial | *tup1* | **strong** | *gpa2* |
| partial | *hmx1* | **partial** | *rim20* |
| partial | *tpk1* | **unknown** | *orf19.6739* |
| partial | *ftr1* | **unknown** | *cdc19* |
| unknown | *nag3* | **unknown** | *ino1* |
| unknown | *sod2* |  |  |
| unknown | *bgl2* |  |  |

| relationship | Zebrafish  iron-related immune protein | relationship | Zebrafish  glucose-related immune protein |
| --- | --- | --- | --- |
| strong | *alas2* | **partial** | *dbh* |
| strong | *hamp1* | **partial** | *gpia* |
| partial | *glrx5* | **partial** | *hif1ab* |
| partial | *slc25a37* | **partial** | *onecutl* |
| partial | *slc40a1* | **partial** | *thbs1* |
| partial | *hpx* | **unknown** | *pgm3* |
| partial | *jmjd6* | **unknown** | *src* |
| partial | *ndfip1* |  |  |
| unknown | *src* |  |  |
| unknown | *mb* |  |  |
| unknown | *sod2* |  |  |

**Table 2. We list the statistically significant proteins identified in the invasion stage with C. albicans and zebrafish as well as iron- and glucose-related classification.**

**Damage stage**

**Differentially regulated proteins with strong evidence**

In *C. albicans*, we identified *hem3*, *tpk2,* and *cph1* from the iron host-pathogen interspecies interaction network in the damage stage. *Hem3* is known to be related to *C. albicans* pathogenesis [[14](#_ENREF_72)]. *Tpk2* is reported to be essential for full virulence and filamentous growth [[15](#_ENREF_62)]. *Cph1* is reported to be involved in both filamentous growth and pathogenesis, as *cph1* mutation renders *C. albicans* unable to form hyphae or penetrate the chorioallantoic membrane [[16](#_ENREF_73)].

For the glucose interaction network in the damage stage, *cdc24*, *gsc1*, and *tpk2* were identified. It is reported that *cdc24* is responsible for invasive hyphal growth and is also required for the pathogenicity of *C. albicans* [[17](#_ENREF_74)]. *Gsc1* is reported to be essential for *C. albicans* virulence and is involved in glutathione synthesis. Knock-out of *gsc1* leads to reduced virulence in mice [[18](#_ENREF_75)]. The identification of these proteins in the damage stage confirms the effectiveness of our proposed method.

| relationship | *C. albicans*  iron-related virulence protein | relationship | *C. albicans*  glucose-related virulence protein |
| --- | --- | --- | --- |
| strong | *hem3* | **strong** | *cdc24* |
| strong | *tpk2* | **strong** | *gsc1* |
| strong | *cph1* | **strong** | *tpk2* |
| partial | *fre10* | **unknown** | *cdc19* |
| partial | *tup1* |  |  |
| partial | *phr2* |  |  |
| partial | *hap43* |  |  |
| partial | *ftr2* |  |  |
| unknown | *mig1* |  |  |

| relationship | Zebrafish  iron-related immune protein | relationship | Zebrafish  glucose-related immune protein |
| --- | --- | --- | --- |
| strong | *alas2* | **partial** | *edn1* |
| strong | *hamp1* | **unknown** | *ptenb* |
| partial | *slc40a1* |  |  |
| partial | *tfr1a* |  |  |
| partial | *hpx* |  |  |
| partial | *jmjd6* |  |  |

**Table 3. We list the statistically significant proteins identified in the damage stage with C. albicans and zebrafish as well as iron- and glucose-related classification.**

**Reference**

1. Villar CC, Kashleva H, Dongari-Bagtzoglou A (2004) Role of Candida albicans polymorphism in interactions with oral epithelial cells. Oral Microbiol Immunol 19: 262-269.

2. Kim SW, Joo YJ, Kim J (2010) Asc1p, a ribosomal protein, plays a pivotal role in cellular adhesion and virulence in Candida albicans. J Microbiol 48: 842-848.

3. Brown V, Sexton JA, Johnston M (2006) A glucose sensor in Candida albicans. Eukaryot Cell 5: 1726-1737.

4. Miwa T, Takagi Y, Shinozaki M, Yun CW, Schell WA, et al. (2004) Gpr1, a putative G-protein-coupled receptor, regulates morphogenesis and hypha formation in the pathogenic fungus Candida albicans. Eukaryot Cell 3: 919-931.

5. Vyas VK, Kuchin S, Berkey CD, Carlson M (2003) Snf1 kinases with different beta-subunit isoforms play distinct roles in regulating haploid invasive growth. Mol Cell Biol 23: 1341-1348.

6. Brownlie A, Donovan A, Pratt SJ, Paw BH, Oates AC, et al. (1998) Positional cloning of the zebrafish sauternes gene: a model for congenital sideroblastic anaemia. Nat Genet 20: 244-250.

7. Shike H, Shimizu C, Lauth X, Burns JC (2004) Organization and expression analysis of the zebrafish hepcidin gene, an antimicrobial peptide gene conserved among vertebrates. Dev Comp Immunol 28: 747-754.

8. Stoldt VR, Sonneborn A, Leuker CE, Ernst JF (1997) Efg1p, an essential regulator of morphogenesis of the human pathogen Candida albicans, is a member of a conserved class of bHLH proteins regulating morphogenetic processes in fungi. EMBO J 16: 1982-1991.

9. Cintia R. C. Rocha KS, Doreen Harcus, Anne Marcil, Daniel Dignard, Brad N. Taylor, David Y. Thomas, Malcolm Whiteway, and Ekkehard Leberer (2001) Signaling through adenylyl cyclase is essential for hyphal growth and virulence in the pathogenic fungus Candida albicans.

10. Bai C, Xu XL, Wang HS, Wang YM, Chan FY, et al. (2011) Characterization of a hyperactive Cyr1 mutant reveals new regulatory mechanisms for cellular cAMP levels in Candida albicans. Mol Microbiol 82: 879-893.

11. Sims GP, Aitken R, Rogerson A (2002) Identification and phylogenetic analysis of morphologically similar naked amoebae using small subunit ribosomal RNA. J Eukaryot Microbiol 49: 478-484.

12. Zaragoza O, Blazquez MA, Gancedo C (1998) Disruption of the Candida albicans TPS1 gene encoding trehalose-6-phosphate synthase impairs formation of hyphae and decreases infectivity. J Bacteriol 180: 3809-3815.

13. Sanchez-Martinez C, Perez-Martin J (2002) Gpa2, a G-protein alpha subunit required for hyphal development in Candida albicans. Eukaryot Cell 1: 865-874.

14. Shepherd MG (1985) Pathogenicity of morphological and auxotrophic mutants of Candida albicans in experimental infections. Infect Immun 50: 541-544.

15. Stichternoth C, Fraund A, Setiadi E, Giasson L, Vecchiarelli A, et al. (2011) Sch9 kinase integrates hypoxia and CO2 sensing to suppress hyphal morphogenesis in Candida albicans. Eukaryot Cell 10: 502-511.

16. Gow NA, Knox Y, Munro CA, Thompson WD (2003) Infection of chick chorioallantoic membrane (CAM) as a model for invasive hyphal growth and pathogenesis of Candida albicans. Med Mycol 41: 331-338.

17. Bassilana M, Blyth J, Arkowitz RA (2003) Cdc24, the GDP-GTP exchange factor for Cdc42, is required for invasive hyphal growth of Candida albicans. Eukaryot Cell 2: 9-18.

18. Yadav AK, Desai PR, Rai MN, Kaur R, Ganesan K, et al. (2011) Glutathione biosynthesis in the yeast pathogens Candida glabrata and Candida albicans: essential in C. glabrata, and essential for virulence in C. albicans. Microbiology 157: 484-495.
